# The ATM Signaling Cascade Promotes Recombination-Dependent Pachytene Arrest in Mouse Spermatocytes

**DOI:** 10.1371/journal.pgen.1005017

**Published:** 2015-03-13

**Authors:** Sarai Pacheco, Marina Marcet-Ortega, Julian Lange, Maria Jasin, Scott Keeney, Ignasi Roig

**Affiliations:** 1 Genome Integrity and Instability Group, Institut de Biotecnologia i Biomedicina, Universitat Autònoma de Barcelona, Cerdanyola del Vallès, Spain; 2 Cytology and Histology Unit, Department of Cell Biology, Physiology and Immunology, Universitat Autònoma de Barcelona, Cerdanyola del Vallès, Spain; 3 Molecular Biology Program, Memorial Sloan Kettering Cancer Center, New York, New York, United States of America; 4 Developmental Biology Program, Memorial Sloan Kettering Cancer Center, New York, New York, United States of America; 5 Howard Hughes Medical Institute, Memorial Sloan Kettering Cancer Center, New York, New York, United States of America; National Cancer Institute, UNITED STATES

## Abstract

Most mutations that compromise meiotic recombination or synapsis in mouse spermatocytes result in arrest and apoptosis at the pachytene stage of the first meiotic prophase. Two main mechanisms are thought to trigger arrest: one independent of the double-strand breaks (DSBs) that initiate meiotic recombination, and another activated by persistent recombination intermediates. Mechanisms underlying the recombination-dependent arrest response are not well understood, so we sought to identify factors involved by examining mutants deficient for TRIP13, a conserved AAA+ ATPase required for the completion of meiotic DSB repair. We find that spermatocytes with a hypomorphic *Trip13* mutation (*Trip13^mod/mod^*) arrest with features characteristic of early pachynema in wild type, namely, fully synapsed chromosomes without incorporation of the histone variant H1t into chromatin. These cells then undergo apoptosis, possibly in response to the arrest or in response to a defect in sex body formation. However, TRIP13-deficient cells that additionally lack the DSB-responsive kinase ATM progress further, reaching an H1t-positive stage (i.e., similar to mid/late pachynema in wild type) despite the presence of unrepaired DSBs. TRIP13-deficient spermatocytes also progress to an H1t-positive stage if ATM activity is attenuated by hypomorphic mutations in *Mre11* or *Nbs1* or by elimination of the ATM-effector kinase CHK2. These mutant backgrounds nonetheless experience an apoptotic block to further spermatogenic progression, most likely caused by failure to form a sex body. DSB numbers are elevated in *Mre11* and *Nbs1* hypomorphs but not *Chk2* mutants, thus delineating genetic requirements for the ATM-dependent negative feedback loop that regulates DSB numbers. The findings demonstrate for the first time that ATM-dependent signaling enforces the normal pachytene response to persistent recombination intermediates. Our work supports the conclusion that recombination defects trigger spermatocyte arrest via pathways than are genetically distinct from sex body failure-promoted apoptosis and confirm that the latter can function even when recombination-dependent arrest is inoperative. Implications of these findings for understanding the complex relationships between spermatocyte arrest and apoptosis are discussed.

## Author Summary

Meiosis is the specialized cell division by which haploid cells are produced. As germ cells enter the first meiotic prophase, programmed double-stranded breaks (DSBs) are formed throughout the genome. Repair of these DSBs by homologous recombination is crucial for proper segregation of homologous chromosomes at the end of the first meiotic division, and thus, for the production of haploid gametes. Moreover, failure to correctly repair these DSBs can have deleterious effects on the genomic integrity of offspring. To ensure that meiocytes that fail to repair meiotic DSBs do not complete meiosis, recombination is tightly controlled. However, the signaling pathway(s) tying meiotic recombination to meiotic progression in mouse spermatocytes is not known. We report here that the ATM-signaling pathway, composed of the MRE11 complex, ATM and CHK2, is responsible for activation of the recombination-dependent arrest that occurs in *Trip13* mutant mouse spermatocytes, which accumulate unrepaired DSBs during meiotic prophase.

## Introduction

Meiosis generates haploid cells from a diploid progenitor by coupling one round of genome replication to two rounds of chromosome segregation. During prophase of the first division, SPO11 protein forms double-strand breaks (DSBs), whose repair enables homologous chromosomes to pair, synapse and recombine [1]. DSB repair failure has deleterious effects, so meiotic recombination is monitored to ensure its completion [2–8]. When recombination intermediates persist, a system often referred to as the pachytene checkpoint is activated to delay or stop meiotic progression or, in some cases, to initiate programmed cell death [9]. In this paper, we use the term “checkpoint” in the well-established sense of a signaling mechanism that creates a relationship (dependency) between two otherwise independent meiotic processes [8]. In this context, checkpoint responses to a cellular defect could have any of several non-exclusive downstream consequences including arrested differentiation, cell cycle arrest, and/or programmed cell death.

Mouse spermatocytes unable to complete recombination (e.g., if they lack the strand-exchange protein DMC1 [10,11]) arrest differentiation without expressing the testis-specific histone variant H1t [2], which is a marker of mid-pachynema and later stages of spermatogenesis [12,13]. In contrast, spermatocytes unable to make DSBs at all (i.e., in the absence of SPO11 [14,15]) are able to progress to a state where H1t is expressed [2] (See S1 Table for a more detailed summary of the phenotypes of these and other mouse mutants used in this study). Thus, male mammalian meiocytes respond differently to the inability to complete recombination once it has begun as opposed to the absence of recombination initiation. In both types of mutant, however, spermatocytes undergo apoptosis during pachynema [16]. It is now clear that one DSB-independent trigger of spermatocyte apoptosis involves failure to form a sex body [16], the transcriptionally repressed, heterochromatic domain encompassing the nonhomologous portions of the X and Y chromosomes. Sex body failure enables expression of sex chromosome genes that for unknown reasons are deleterious for pachytene spermatocytes [17]. This cell elimination mechanism depends on the checkpoint kinase ATR [18]. We will refer to spermatocyte arrest in response to DSB repair defects, measured as a block to H1t expression, as *recombination-dependent arrest* to distinguish it from the DSB-independent *sex body-deficient arrest*.

It has been suggested that both types of arrest are sufficient to trigger apoptosis [10,14,15], although this remains unproven for recombination-dependent arrest. Interestingly, however, even though spermatocytes arrest with physiological states characteristic of different points in prophase depending on the type of recombination defect, they undergo apoptosis at an equivalent stage when evaluated in the context of development of the seminiferous epithelium [2,19]. Spermatogenesis occurs within the seminiferous tubules, which can be classified among twelve epithelial stages (I to XII) according to the cell types found in each cross-section [20]. Despite showing different arrest points based on H1t staining, *Dmc1* ^−/−^ and *Spo11* ^−/−^ spermatocytes undergo apoptosis at the same epithelial stage (stage IV), which is the equivalent of mid-pachynema for wild-type spermatocytes [2,19], and which is the time when H1t incorporation would normally have occurred [13] (described in more detail below). The inability of epithelial staging alone to discriminate between different physiological arrest points emphasizes the importance of molecular cytological methods for characterizing mutant spermatocyte behavior [2]. A straightforward interpretation is that recombination-dependent arrest blocks spermatogenic differentiation at a stage equivalent to early pachynema, with cells remaining in this arrested state until apoptosis is triggered in epithelial stage IV, equivalent to the time unarrested cells would have transitioned into mid-pachynema.

Exploring mechanisms unique to recombinant-dependent arrest is challenging: meiotic recombination drives pairing and synapsis of homologous chromosomes in mouse, so most mutants that fail to complete recombination also show widespread chromosome asynapsis, which indirectly blocks sex body formation [16]. Thus, mutants like *Dmc1* that retain numerous unrepaired DSBs at early pachynema also display rampant chromosomal asynapsis, which impedes sex body formation [2,16]. Interpreting this arrest phenotype is further complicated if asynapsis *per se* can trigger a response separate from its effects on sex body formation [21]. Since these mutants have such a complex array of severe defects, it is difficult to use them to explore mechanisms unique to recombination-dependent arrest. Thus, we wished to use mutants that cannot complete DSB repair but that do complete synapsis.

Two mutants are known to enter pachynema with unrepaired DSBs yet with substantially normal autosomal synapsis and apparently normal sex body formation, providing an opportunity to investigate factors that specifically regulate recombination-dependent arrest during meiosis. One mutant is *Trip13^mod/mod^*, which contains a hypomorphic gene trap allele of *Trip13* (*Trip13^mod^*, for “moderately defective”; also termed *Trip13^Gt^*) [22,23]. This allele substantially reduces expression of TRIP13, a AAA+ ATPase required for meiotic recombination. Most *Trip13^mod/mod^* spermatocytes arrest at pachynema with unrepaired DSBs and complete autosomal synapsis, then subsequently undergo apoptosis [22,23]. We show here that arrest occurs prior to histone H1t incorporation, similar to *Dmc1^−/−^* mutants. A small fraction of cells manage to evade arrest and finish meiosis (“escapers”), presumably because they complete meiotic recombination [22,23]. Nonetheless, the great majority of TRIP13-deficient spermatocytes undergo what has been inferred to be a recombination-dependent arrest in pachynema since autosomal synapsis is completed and spermatocytes display apparently normal looking sex bodies [22,23].

The second mutant is the *Spo11^+/−^* *Atm* ^−/−^ mouse [24,25]. In somatic cells, the ATM kinase is activated by the MRE11 complex to participate in the DNA damage response by phosphorylating multiple substrates, including the checkpoint mediator kinase CHK2 [26]. In meiosis, ATM regulates SPO11 activity via a negative feedback loop, such that *Atm* ^−/−^ single mutant spermatocytes experience greatly elevated DSB levels [27]. As a result, cells are unable to repair DSBs sufficiently and thus fail to complete synapsis or form sex bodies, arresting at pachynema [28]. However, introduction of *Spo11* heterozygosity reduces SPO11 activity, partially decreasing DSB numbers, and so suppresses some of the phenotype of *Atm* ^−/−^ spermatocytes [27]. Although *Spo11^+/−^* *Atm^−/−^* spermatocytes are unable to repair all DSBs at pachynema, they complete autosome synapsis and sex body formation [24,25].

Remarkably, unlike *Trip13* ^*mod/mod*^ spermatocytes, *Spo11* ^*+/−*^ *Atm* ^−/−^ spermatocytes do not arrest or undergo apoptosis at pachynema despite the presence of multiple unrepaired DSBs, instead progressing to metaphase I before initiating programmed cell death [24,25]. To explain this difference in arrest behavior, we hypothesized that ATM is a critical component of the recombination-dependent arrest mechanism. Consistent with this idea, the canonical ATM-effector kinase CHK2 is required to arrest *Trip13* ^*mod/mod*^ oocytes [29]. Here we test this hypothesis by performing epistasis experiments combining *Trip13* ^*mod/mod*^ with mutations that eliminate ATM entirely, attenuate ATM activity, or eliminate a downstream effector kinase. Our findings reveal that recombination-dependent arrest in meiosis prior to the H1t-positive substage of pachynema depends on the MRE11 complex, ATM and CHK2.

## Results

### Different arrest and apoptosis responses in pachynema in *Trip13* ^*mod/mod*^ and *Spo11* ^*+/−*^ *Atm^−/−^* spermatocytes despite persistence of unrepaired DSBs in both genotypes

It has been proposed that the arrest and subsequent apoptosis of *Trip13* ^*mod/mod*^ spermatocytes at pachynema are both consequences of unrepaired DSBs activating a recombination-dependent checkpoint arrest mechanism [16,22,23]. To test this hypothesis, we asked if *Trip13* ^*mod/mod*^ cells with unrepaired DSBs were indeed undergoing apoptosis while retaining developmental characteristics of early pachynema. We detected apoptotic cells by performing TUNEL staining on spermatocyte spreads previously immunostained for three markers: γH2AX, a phosphorylated form of histone variant H2AX that is a marker of DSBs and sex body formation [30]; SYCP3, a component of the synaptonemal complex [31]; and H1t. For *Spo11* ^−/−^ spermatocytes, in which sex body failure triggers arrest and apoptosis after H1t incorporation has begun [2], all TUNEL-positive cells were also positive for H1t staining (n = 59, S1A Fig.). By contrast, for *Dmc1* ^−/−^ spermatocytes, all TUNEL-positive cells were H1t-negative (n = 70, P≤0.0001, Fisher’s exact test, S1B Fig.). These results further validate use of H1t as a molecular marker to distinguish between different arrest points in meiotic prophase.

**Fig 1:**
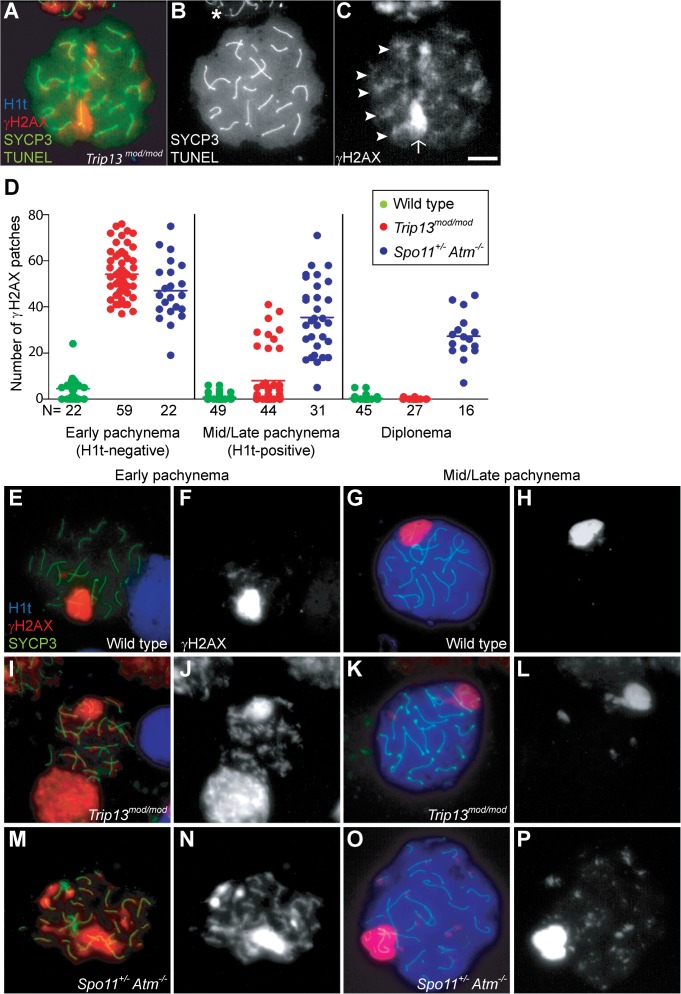
*Trip13* ^*mod/mod*^ spermatocytes activate a recombination-dependent arrest at pachynema. (A) Apoptotic *Trip13* ^*mod/mod*^ spermatocyte showing the axial element protein SYCP3 (green), TUNEL (green), mid/late pachytene stage marker H1t (blue) and γH2AX staining (red). Absence of H1t staining (blue) in the TUNEL-positive cell indicates that this is an early pachytene stage spermatocyte. (B) TUNEL staining appears as pan-nuclear labelling that overlaps all chromatin, which is observed in only a fraction of the analysed cells (asterisk shows an adjacent TUNEL-negative cell). (C) γH2AX localizes to the sex body (arrow) and unrepaired breaks (arrowheads). (D) Number of autosomal γH2AX patches (similar to arrowheads in (C)) counted in H1t-negative (early) and H1t-positive (mid/late) pachytene and diplotene cells from animals of the indicated genotypes. Means are indicated with horizontal lines. N shows the total number of cells counted per each stage and genotype. Primary data are provided in S1 Dataset. (E-P) Representative cells from the indicated genotypes stained for H1t (blue), γH2AX (red) and SYCP3 (green). In *Trip13* ^*mod/mod*^, mid/late pachytene cells have substantially fewer γH2AX patches than early pachytene spermatocytes. Bar in (C) represents 10 μm and applies to all panels.

Nearly all *Trip13* ^*mod/mod*^ TUNEL-positive cells (97.2%, n = 106) were H1t-negative (Fig. 1A-B), implying that most apoptotic cells had arrested in a developmental state with characteristics of early pachynema, as predicted. All *Trip13* ^*mod/mod*^ TUNEL-positive spermatocytes displayed multiple γH2AX patches (Fig. 1C), confirming the presence of unrepaired DSBs. A grossly normal sex body was also observed, agreeing with prior results [22,23], although further analysis described below revealed subtle sex body defects.

If recombination-dependent arrest is activated in *Trip13* ^*mod/mod*^ spermatocytes, then we would also predict that the subset of cells that progress further to an H1t-positive state are the relatively recombination-proficient escapers documented previously [22,23]. If so, these cells should have fewer unrepaired DSBs than the pachytene cells that arrested before incorporating H1t. Indeed, H1t-positive *Trip13* ^*mod/mod*^ pachytene cells had fewer γH2AX patches than H1t-negative cells (8.0 ± 12.2 vs. 54.2 ± 9.9, mean ± SD, respectively; P≤0.0001, t test; Fig. 1D-L). Interestingly, there appeared to be two populations of H1t-positive *Trip13* ^*mod/mod*^ pachytene spermatocytes. The majority (77.3%) had very few (< 8) γH2AX patches (1.7 ± 2.2 on average; Fig. 1K-L), but still significantly more than wild-type cells (0.8 ± 1.5; P = 0.016; Fig. 1D, G-H). The remainder (22.7%) had more γH2AX patches than the first population (29.4 ± 6.7; P≤0.0001), but significantly less than H1t-negative cells (P≤0.0001). For the few *Trip13* ^*mod/mod*^ cells that reached diplonema, however, the number of γH2AX patches was indistinguishable from wild type (0.1 ± 0.3 vs. 0.4 ± 1.1, respectively; P = 0.183, negative binomial regression; Fig. 1D). A straightforward interpretation is that stochastic differences between TRIP13-deficient cells in the number of unrepaired DSBs translate into different arrest responses: cells with many unrepaired DSBs experience recombination-dependent arrest at early pachynema (H1t-negative), cells with an intermediate number progress to an H1t-positive stage but then undergo apoptosis (possibly because of the DSBs, sex body defects, or both; see below), and the most repair-proficient subset of cells progress still further to diplonema. Additional implications of these patterns are addressed in Discussion.

Spermatocytes from *Spo11* ^*+/−*^ *Atm* ^−/−^ mice also display γH2AX patches late in prophase I yet progress to the first division; this difference from ATM-proficient *Trip13* ^*mod/mod*^ mice is consistent with the hypothesis that cells respond differently to persistent DSBs in the absence of ATM. Using H1t staining to more precisely define prophase stages, we observed that H1t-positive *Spo11* ^*+/−*^ *Atm* ^−/−^ spermatocytes had numerous γH2AX patches, only ∼20% fewer than H1t-negative *Spo11* ^*+/−*^ *Atm* ^−/−^ spermatocytes (Fig. 1D, M-P). This is in marked contrast to *Trip13* ^*mod/mod*^ mice, where most cells that progressed to an H1t-positive state had much fewer γH2AX patches than the earlier H1t-negative cells (see above). Moreover, H1t-positive *Spo11* ^*+/−*^ *Atm* ^−/−^ spermatocytes had significantly more γH2AX patches than total H1t-positive *Trip13* ^*mod/mod*^ cells (35.5 ± 15.6, P≤0.0001; Fig. 1D). Thus, in the absence of ATM, cells with numerous unrepaired DSBs can progress to mid/late pachynema (and beyond).

### *Trip13* ^*mod/mod*^ *Spo11* ^*+/−*^ *Atm^−/−^* spermatocytes progress to an H1t-positive state in greater numbers despite a high level of persistent DSBs

The above results are consistent with our hypothesis that unrepaired DSBs at early pachynema in *Trip13* ^*mod/mod*^ spermatocytes trigger a recombination-dependent checkpoint arrest that is mediated by ATM. To test this idea, we wished to ask if removing ATM activity allows TRIP13-deficient cells to progress further into prophase. It is uninformative for this purpose to query *Trip13* ^*mod/mod*^ *Atm^−/−^* double mutants because the *Atm^−/−^* mutation by itself causes an early block at an H1t-negative stage as a consequence of the enormous (>10-fold) increase in DSB numbers (see Discussion) [2,27]. Instead, since *Spo11* heterozygosity ameliorates this catastrophic effect of ATM deficiency, we tested the epistasis relationship between the *Trip13* ^*mod/mod*^ phenotype (arrest in an H1t-negative state) and *Spo11* ^*+/−*^ *Atm^−/−^* phenotype (no pachytene arrest) by analyzing *Trip13* ^*mod/mod*^ *Spo11* ^*+/−*^ *Atm* ^−/−^ mice (hereafter referred to as “TSA triple mutant”). As detailed below, we indeed find that absence of ATM allows TRIP13-deficient spermatocytes to progress to an H1t-positive state, but that additional defects that cause efficient mid/late-pachytene arrest and apoptosis of TSA triple mutant spermatocytes become apparent. (Note that DSBs in the *Spo11* ^*+/−*^ *Atm^−/−^* background are still substantially elevated relative to wild type (∼6-fold) [27], so no aspect of the triple mutant phenotype can be ascribed to a reduction in DSBs below wild-type levels.)

First, we measured testis size as a readout of spermatogenetic progression since mice with spermatogenic failure have small testes [14,15]. Sizes of *Trip13* ^*mod/mod*^ and TSA triple mutant testes were indistinguishable from one another (P = 0.6, t test), both being smaller than in *Spo11* ^*+/−*^ *Atm* ^−/−^ mice (P≤0.05, Table 1) and approximately one third the size of wild-type testes (P≤0.0001, Table 1). These results indicate that TSA triple mutant mice experience spermatogenic failure.

**Table 1:** Testicular and meiotic phenotypes of wild-type and mutant mice.

	Wild type	*Trip13* ^*mod/mod*^	*Spo11* ^*+/−*^ *Atm* ^–/–^	*Trip13* ^*mod/mod*^ *Spo11* ^*+/−*^ *Atm* ^–/–^	*Mre11* ^*ATLD/ATLD*^	*Trip13* ^*mod/mod*^ *Mre11* ^*ATLD/ATLD*^	*Nbs1* ^*ΔB/ΔB*^	*Trip13* ^*mod/mod*^ *Nbs1* ^*ΔB/ΔB*^	*Chk2* ^–/–^	*Trip13* ^*mod/mod*^ *Chk2* ^-l/-^
N.T.W. (mean ± SEM)	0.66 ± 0.03	0.20 ± 0.01	0.36 ± 0.04	0.21 ± 0.05	0.65 ± 0.05	0.19 ± 0.02	0.51^a^	0.21 ± 0.02	0.59 ± 0.1	0.19 ± 0.02
Epithelial Arrest	No arrest	IV (escapers)	XII (escapers)	IV (no escapers)	No arrest	IV (no escapers)	No arrest	IV (no escapers)	No arrest	IV (escapers)
% Tubules with ˃5 TUNEL-positive cells (N)	1.0 ^a^ (100)	21.6 ^a^ (102)	11.9 (101)	26.0 ^a^ (100)	7.0 ^a^ (100)	16.0 ^a^ (100)	10.0 ^a^ (100)	25.0 ^a^ (100)	3.0 ^a^ (100)	16.0 ^a^ (100)
% cells Post-pachynema (N)	31.6 (973)	3.3 (705)	15.6 (700)	0.0 (703)	25.2 (309)	0.0 (348)	49.8 (211)	0.3 (350)	18.1 (299)	1.9 (360)
% cells H1t positive (N)	50.3*(350)	20.8 (567)	45.8* (500)	47.7* (532)	61.06* (208)	73.3* (221)	64.0* (100)	43.4* (219)	57.0* (100)	46.0* (237)

N.T.W.: Normalized Testis Weight expressed as a percentage of body weight.

^a^: one mouse analysed.

*: Significantly different from what is found in *Trip13* ^*mod/mod*^ mice, P˂0.05, Fisher’s exact test.

**Fig 2:**
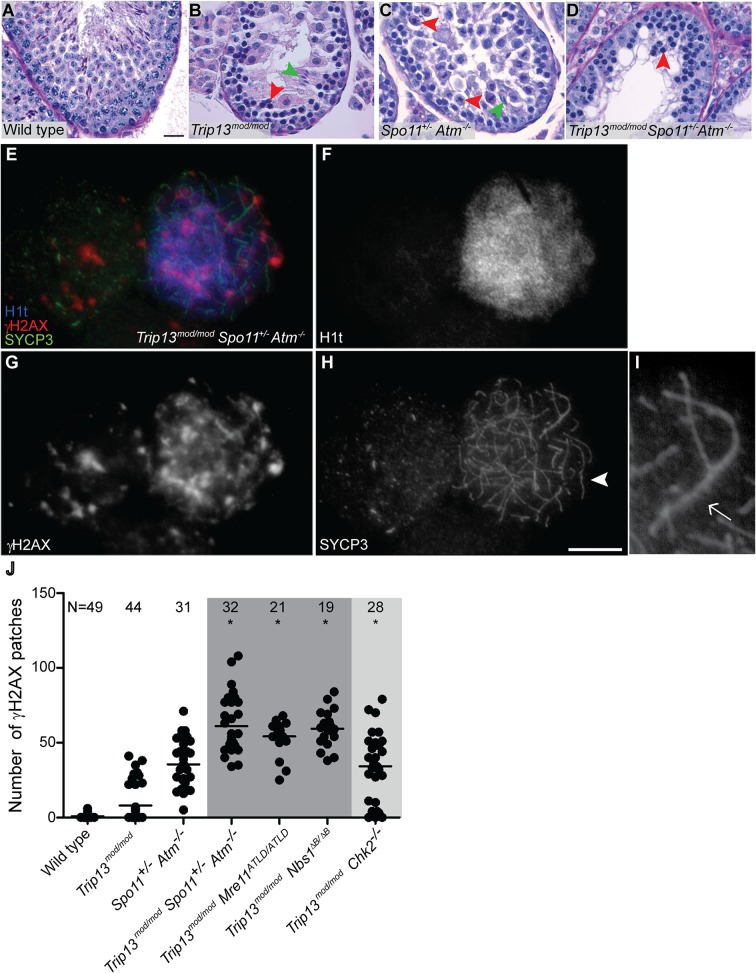
*Trip13* ^*mod/mod*^ *Spo11* ^*+/−*^ *Atm* ^−/−^ spermatocytes arrest at epithelial stage IV, but present autosomal asynapsis, multiple unrepaired DSBs and fail to form a sex body at mid/late pachynema. (A-D) Testis cross-sections showing individual seminiferous tubules from the indicated genotypes, stained with PAS-Haematoxylin. (A) In wild type, spermatogonia, spermatocytes and spermatids are present. (B) Stage IV tubule in *Trip13* ^*mod/mod*^ with apoptotic spermatocytes, corresponding to pachynema (red arrowhead) [22,23]. Only a minor fraction of cells complete meiosis (green arrowhead). (C) *Spo11* ^*+/−*^ *Atm* ^−/−^ tubule at stage XII, which is characterized by spermatocytes at metaphase I (red arrowhead) [24]. The presence of lagging chromosomes in some metaphase I spermatocytes is thought to be the cause of apoptosis. Green arrowhead denotes a spermatid that has escaped meiotic arrest. (D) *Trip13* ^*mod/mod*^ *Spo11* ^*+/−*^ *Atm* ^−/−^ tubule at stage IV, presenting multiple apoptotic spermatocytes at pachytene stage (red arrowhead). No spermatids were observed. (E-H) Two *Trip13* ^*mod/mod*^ *Spo11* ^*+/−*^ *Atm* ^−/−^ spermatocytes, one at preleptonema (left) and the other at mid/late pachynema (right), stained for H1t (blue, F), γH2AX (red, G) and SYCP3 (green, H). A high degree of asynapsis occurs in the H1t-positive cell, where long stretches of axial elements are visible (arrowhead). (I) Enlarged image of a bivalent that has successfully synapsed only a portion of the homologous chromosome pair (arrow). (J) Number of γH2AX patches in mid/late pachytene spermatocytes of the indicated genotypes. Triple and double mutants that generate more DSBs and fail to complete synapsis (highlighted in dark grey) display more γH2AX patches at mid/late pachynema than *Trip13* ^*mod/mod*^ *Chk2* ^−/−^ cells (highlighted in light grey). Data for wild type, *Trip13* ^*mod/mod*^ and *Spo11* ^*+/−*^ *Atm* ^−/−^ reproduced from Fig. 1D for comparison. N shows the total number of cells counted per each genotype. Asterisk marks statistically significant differences compared to *Trip13* ^*mod/mod*^, P≤0.0001, t test. Primary data are provided in S1 Dataset. Bar in (A) represents 20 μm and applies to panels (A-D). Bar in (H) represents 10 μm and applies to panels (E-H).

Second, we assessed the timing of apoptosis by histological staging of seminiferous tubules. Many *Trip13* ^*mod/mod*^ spermatocytes underwent apoptosis in tubules at epithelial stage IV, corresponding to mid pachynema (Fig. 2A,B), as previously shown [23]. In contrast, and consistent with absence of pachytene arrest, *Spo11* ^*+/−*^ *Atm* ^−/−^ spermatocytes apoptosed in tubules at epithelial stage XII (Fig. 2C), corresponding to metaphase I. Arrest of *Spo11* ^*+/−*^ *Atm^−/−^* spermatocytes at this point is thought to be caused largely by a spindle checkpoint response to achiasmate (unconnected) chromosomes, particularly the X-Y pair [32]. Consistent with the small testis sizes, TSA triple mutant animals showed spermatocyte apoptosis at stage IV (Fig. 2D). Furthermore, whereas few wild-type tubules had >5 TUNEL-positive cells (1.0%, Table 1), both *Trip13* ^*mod/mod*^ single mutant and TSA triple mutant testes displayed many such apoptotic tubules (21.6% and 26.0% respectively, P≤0.0001 compared to wild type, Fisher’s exact test, Table 1). Surprisingly, no TSA triple mutant cells escaping stage IV apoptosis were observed (Fig. 2B-D), in contrast to *Trip13* ^*mod/mod*^ or *Spo11* ^*+/−*^ *Atm* ^−/−^ testes [22–24].

Although these results indicate that ATM deficiency (in the *Spo11* ^*+/−*^ *Atm^−/−^* background) does not suppress the pachytene apoptosis of *Trip13* ^*mod/mod*^ mutant spermatocytes, they do not precisely define the stage of meiotic arrest. To address this question, we examined progression of chromosome synapsis in spermatocyte spreads stained with anti-SYCP3 antibodies. Out of total SYCP3-positive primary spermatocytes, 3.3% had progressed beyond pachynema in *Trip13* ^*mod/mod*^, but none in TSA triple mutants (P≤0.0001, Fisher’s exact test, Table 1). This lack of escapers here and in the analysis of tubule sections indicates that overall spermatogenic failure is more penetrant in TSA triple mutants. Moreover, all TSA triple mutant spermatocytes displayed substantial autosome asynapsis (n = 703, Fig. 2E, H-I), unlike either the *Trip13* ^*mod/mod*^ or *Spo11* ^*+/–*^ *Atm^−/−^* mutants [23,24]. Because failure to complete synapsis has been associated with the inability to properly repair meiotic DSBs, these results could suggest that TRIP13-deficient spermatocytes are unable to cope with the increased DSB numbers produced in the *Spo11* ^*+/−*^ *Atm* ^−/−^ background [27], leading to synaptic catastrophe.

One consequence of synaptic failure is the inhibition of sex body formation [16]. Accordingly, we did not observe any TSA triple mutant cells with the γH2AX immunostaining pattern diagnostic of sex body formation (Fig. 2E, G). Instead, γH2AX signal was mostly localized as patches on both unsynapsed and synapsed portions of chromosomes and did not accumulate in any particular region of the nucleus. The complete failure to form sex bodies in TSA triple mutants likely accounts for the highly penetrant arrest in pachynema and apoptosis at stage IV.

To determine whether ATM imposes a recombination-dependent arrest early in pachynema that is relieved in TSA triple mutant spermatocytes, we examined H1t incorporation. In wild type, 50.3% of SYCP3-positive cells were also positive for H1t (Table 1), whereas only 20.8% of SYCP3-positive cells from *Trip13* ^*mod/mod*^ testes were H1t-positive (Table 1). Presumably, most of these H1t-positive cells were escapers that would complete meiosis (see above). Notably, 47.7% of SYCP3-positive cells from TSA triple mutants were H1t-positive (see representative image in S2 Fig.), a ≥two-fold increase compared to *Trip13* ^*mod/mod*^ samples but similar to that in wild-type and in *Spo11* ^*+/−*^ *Atm^−/−^* animals (45.8%, Table 1). Thus, the *Spo11* ^*+/−*^ *Atm^−/−^* combination is epistatic to *Trip13* ^*mod/mod*^ mutation for the ability of spermatocytes to incorporate H1t.

We note that the relatively normal H1t pattern in *Spo11* ^*+/−*^ *Atm^−/−^* animals (S1 Table) and the strong block to H1t incorporation in *Atm^−/−^* single mutants [2] argue against the possibility that ATM is required to prevent premature H1t expression in early pachynema. To confirm this conclusion, we compared timing of H1t expression in testis sections from wild type and TSA triple mutants. In wild type, H1t was first detected in mid-pachytene spermatocytes in stage IV tubules, and was absent from early pachytene spermatocytes in stage I-III tubules, as previously reported [13] (see S3A,B Fig. and its legend). Staging is less precise for immunofluorescently stained tubules of mutants that lack post-meiotic cell types [19]. Nonetheless, we observed that leptotene, zygotene, and most if not all early pachytene spermatocytes were H1t-negative, and that H1t-positive cells did not appear until approximately mid pachynema in TSA triple mutants (i.e., in tubule sections that were likely in stage IV) (see S3C Fig. and its legend). Thus, there is no evidence for substantially premature expression of H1t in ATM-defective spermatocytes.

The increased number of H1t-positive cells implies that the TSA triple mutant spermatocytes are not being restrained by the recombination-dependent arrest mechanism. This further reinforces the conclusion above that the apoptosis occurring in these cells is attributable to the absence of a sex body. If our interpretation is correct, we would expect that absence of the recombination-dependent checkpoint allows spermatocytes to progress to an H1t-positive state even if they have many unrepaired DSBs. Indeed, H1t-positive TSA triple mutant spermatocytes displayed significantly more γH2AX patches than *Trip13* ^*mod/mod*^ cells (61.0 ± 18.8, P≤0.0001, t test; Fig. 2J). The large number of γH2AX patches also supports the conclusion that the TSA triple mutants do not experience a reduction in DSBs relative to *Trip13* ^*mod/mod*^ alone. The finding that absence of ATM allows cells to progress to an H1t-positive state even if they contain numerous unrepaired DSBs indicates that ATM is required for implementation of recombination-dependent arrest at early pachynema.

### The MRE11 complex promotes recombination-dependent arrest

The MRE11 complex (MRE11, RAD50 and NBS1) senses DSBs and promotes their repair, in part by activating ATM [33]. Null mutations of these genes are incompatible with embryonic development, but mice bearing hypomorphic *Nbs1* or *Mre11* mutations that attenuate ATM activation and/or diminish phosphorylation of subsets of ATM targets are viable (*Nbs1* ^*ΔB*^ or *Mre11* ^*atld*^) [34,35]. *Nbs1* ^*ΔB/ΔB*^ and *Mre11* ^*ATLD/ATLD*^ mice are subfertile, with spermatocytes displaying some synaptic defects and accumulating early recombination markers at pachynema, suggesting that the MRE11 complex plays a role in meiotic recombination in mammals [36], as in budding yeast and other organisms [37]. Testis samples from both mutants showed a small elevation in the fraction of spermatocytes that were H1t-positive (Table 1). This could be a consequence of previously documented differences in the overall distribution of meiotic cell types [36], but the important conclusion for purposes of this study is that the mutant spermatocytes are readily able to progress through pachynema and beyond in roughly normal numbers.

To further define the ATM-dependent pathway leading to recombination-dependent arrest, we asked whether these MRE11-complex mutations mimic ATM deficiency with respect to the meiotic progression phenotype of *Trip13* ^*mod/mod*^ spermatocytes. Indeed, similar to the TSA triple mutant, *Trip13* ^*mod/mod*^ *Nbs1* ^*ΔB/ΔB*^ and *Trip13* ^*mod/mod*^ *Mre11* ^*ATLD/ATLD*^ mutants had approximately two-fold (43.4%) and three-fold (73.3%) more H1t-positive spermatocytes, respectively, than *Trip13* ^*mod/mod*^ single mutants (Table 1). Importantly, these mutants progressed despite having high numbers of unrepaired DSBs: H1t-positive spermatocytes in *Trip13* ^*mod/mod*^ *Nbs1* ^*ΔB/ΔB*^ and *Trip13* ^*mod/mod*^ *Mre11* ^*ATLD/ATLD*^ mutants had significantly more γH2AX patches than the *Trip13* ^*mod/mod*^ mutant (59.7 ± 12.4 and 54.1 ± 10.8, respectively; Fig 2J), but similar numbers to the TSA triple mutant (P˃0.05). These hypomorphic MRE11 complex mutations are thus epistatic to *Trip13* ^*mod/mod*^ with respect to the ability of cells to progress to an H1t-positive stage. These results support the hypothesis that the MRE11 complex activates the ATM-mediated recombination-dependent arrest that halts development of most *Trip13* ^*mod/mod*^ single mutant spermatocytes at early pachynema.

**Fig 3:**
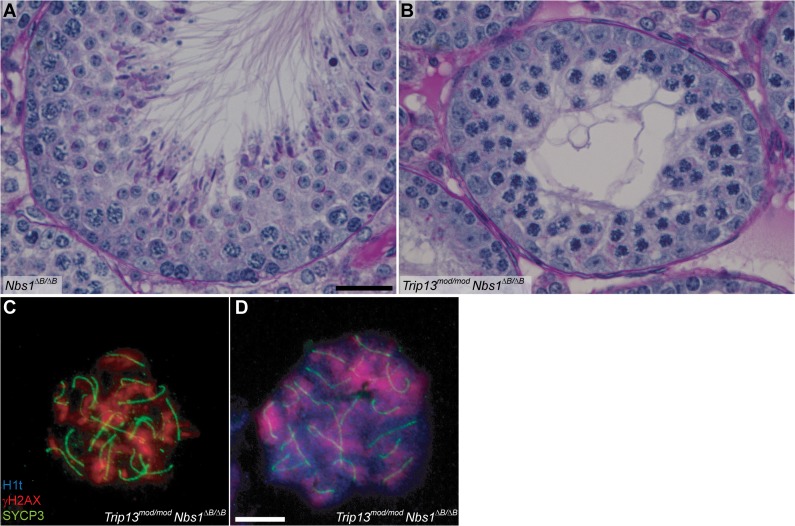
*Trip13* ^*mod/mod*^ *Nbs1* ^*ΔB/ΔB*^ spermatocytes arrest in mid/late pachynema with unrepaired DSBs at epithelial stage IV. (A-B) Cross sections of *Nbs1* ^*ΔB/ΔB*^ and *Trip13* ^*mod/mod*^ *Nbs1* ^*ΔB/ΔB*^ stage IV tubules stained with PAS-Haematoxylin. (C-D) Early (C) and mid/late (D) pachytene *Trip13* ^*mod/mod*^ *Nbs1* ^*ΔB/ΔB*^ spermatocytes stained for H1t, γH2AX, and SYCP3. Both cells present multiple unrepaired DSBs and incomplete synapsis. Bar in (A) represents 20 μm and applies to panels (A-B). Bar in (D) represents 10 μm and applies to panels (C-D).

Also akin to the TSA triple mutant, these double mutants experienced a more penetrant spermatogenic failure (i.e., few or no escapers) than in the *Trip13* ^*mod/mod*^ single mutant despite better progression beyond early pachynema. *Trip13* ^*mod/mod*^ *Nbs1* ^*ΔB/ΔB*^ and *Trip13* ^*mod/mod*^ *Mre11* ^*ATLD/ATLD*^ mice had small testes (Table 1), and histological analysis revealed many similarities between *Trip13* ^*mod/mod*^ *Nbs1* ^*ΔB/ΔB*^ and *Trip13* ^*mod/mod*^ *Mre11* ^*ATLD/ATLD*^ double mutants and the TSA triple mutant: apoptosis at epithelial stage IV (Figs. 3 and S4), a high number of apoptotic cells per tubule (Table 1), and no evidence of cells escaping and completing meiosis (Figs 3. and S4). Likewise, cytological analysis showed that no *Trip13* ^*mod/mod*^ *Mre11* ^*ATLD/ATLD*^ spermatocytes and only a negligible fraction of *Trip13* ^*mod/mod*^ *Nbs1* ^*ΔB/ΔB*^ spermatocytes (0.3%) progressed beyond pachynema, similar to the TSA triple mutant (P˃0.05, Fisher’s exact test, Table 1) but significantly different from the *Trip13* ^*mod/mod*^ single mutant (P≤0.0001 and P = 0.0014, respectively). Also similar to TSA triple mutants, most double mutant spermatocytes had substantial synaptic defects, with only 0.5% of *Trip13* ^*mod/mod*^ *Mre11* ^*ATLD/ATLD*^ (n = 348) and 5.4% of *Trip13* ^*mod/mod*^ *Nbs1* ^*ΔB/ΔB*^ (n = 350) SYCP3-positive spermatocytes showing complete autosomal synapsis. These values are much lower than *Trip13* ^*mod/mod*^ SYCP3-positive spermatocytes (40.7%, n = 705, P≤0.0001, Fisher’s exact test). As expected from the substantial asynapsis, the double mutants showed no evidence of sex body formation as assessed by γH2AX staining (Figs. 3 and S4). Taken together, we infer from these data that the more penetrant spermatogenic failure observed in *Trip13* ^*mod/mod*^ *Nbs1* ^*ΔB/ΔB*^ and *Trip13* ^*mod/mod*^ *Mre11* ^*ATLD/ATLD*^ testes is most probably due to activation of the sex body-deficient arrest, as in TSA triple mutants. Further, our results confirm that the sex body-deficient arrest mechanism can operate even when recombination-dependent arrest is compromised. This was also indicated by the arrest of *Spo11* ^*−/−*^ mutants as well as repair-proficient mutants with defects in meiotic sex chromosome inactivation (MSCI) [2,16].

### The MRE11 complex controls DSB numbers

**Fig 4:**
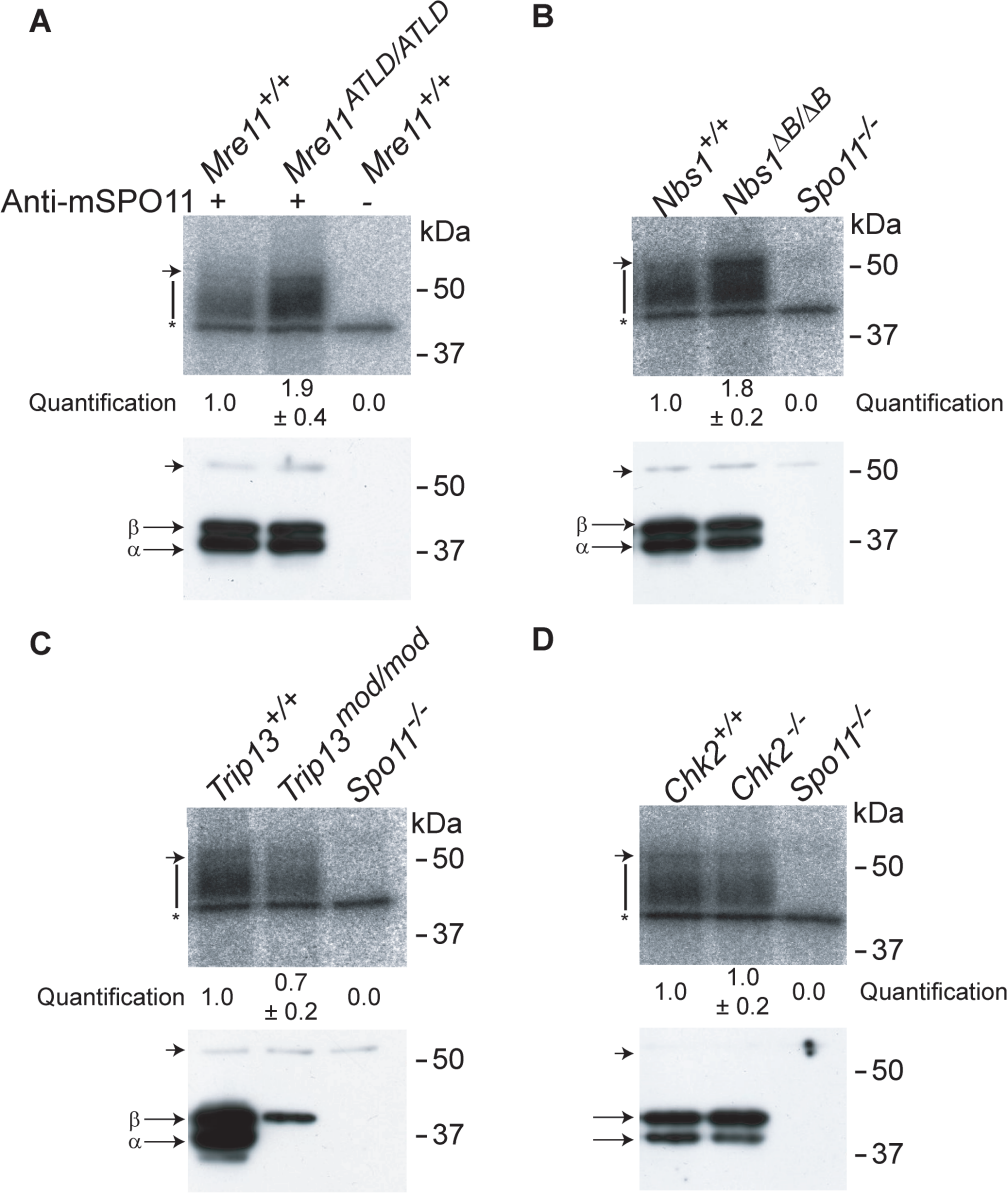
The MRE11 complex, but not TRIP13 or CHK2, modulates SPO11-oligonucleotide complex levels. SPO11-oligonucleotide complexes were immunoprecipitated from extracts of testes of the indicated genotypes, labelled with terminal transferase and ^32^P-nucleotide, and resolved by SDS-PAGE. Top image in each panel, autoradiograph; bottom image, SPO11 Western blot where the two major SPO11 isoforms, α and β, are indicated. The vertical lines next to the autoradiographs indicate the signal from SPO11-oligonucleotide complexes. Asterisk indicates non-specific signal from the labelling reaction. Short arrow designates the migration position of the heavy chain of the antibody used to immunoprecipitate SPO11. (A-B) Both *Mre11* and *Nbs1* mutants have increased levels of SPO11-oligonucleotide complexes compared to littermate controls (1.9 ± 0.4 fold, mean ± SD of the relative signal intensity compared to a wild-type control, n = 3 mice, and 1.8 ± 0.2 fold, n = 3, respectively). (C) SPO11-oligonucleotide complex levels are slightly reduced in *Trip13* ^*mod/mod*^ samples (0.7 ± 0.2, n = 3), similar to other recombination-deficient mutants, such as *Dmc1* ^–/–^ [27]. The SPO11α isoform is not detected in *Trip13* ^*mod/mod*^ testes as observed in other mutants that arrest at pachynema [27]. (D) *Chk2* ^−/−^ testes have similar levels of SPO11-oligonucleotide complex as controls (1.0 ± 0.2 fold, n = 3).

We reasoned that the attenuated ATM signaling in *Mre11* and *Nbs1* hypomorphic mutants might lead to elevated DSB numbers. To test this, we examined SPO11-oligonucleotide complexes, which provide a measure of whole-testis DSB levels, in *Mre11* ^*ATLD/ATLD*^ and *Nbs1* ^*ΔB/ΔB*^ single mutant mice [27]. (Note that this class of hypomorphic MRE11-complex mutation is unlike the *Rad50S* type of mutation, which in yeast blocks endonucleolytic release of SPO11 from DSB ends [33]). Indeed, we found that *Mre11* ^*ATLD/ATLD*^ and *Nbs1* ^*ΔB/ΔB*^ mice displayed an ∼2-fold increase compared to wild-type littermates (Fig. 4A-B). This elevation is less than what is seen in *Atm^–/–^*(∼12-fold) or *Spo11* ^*+/–*^ *Atm^−/−^* testes (∼6-fold), presumably because the *Mre11* and *Nbs1* hypomorphs attenuate but do not eliminate ATM activity [34,35]. (Note that the overall fertility phenotype of the *Mre11* and *Nbs1* single mutants is also significantly milder than for mice lacking ATM (S1 Table)). These results provide strong evidence that ATM-mediated feedback control of DSBs involves an MRE11 complex-dependent ATM activation pathway similar to the response to ionizing radiation. In contrast, *Trip13* ^*mod/mod*^ animals had roughly normal levels of SPO11-oligonucleotide complexes (∼70% of wild type, Fig. 4C). Because *Trip13* ^*mod/mod*^ single mutants have defects in completing DSB repair, we speculate that the autosomal synaptic failure in *Trip13* ^*mod/mod*^ *Mre11* ^*ATLD/ATLD*^ or *Trip13* ^*mod/mod*^ *Nbs1* ^*ΔB/ΔB*^ mice is a synthetic defect caused by an inability of TRIP13-deficient cells to tolerate increased DSB numbers resulting from ATM activation defects. An alternative but not mutually exclusive possibility is that the MRE11 complex and TRIP13 synergistically promote synapsis separately from their effects on recombination.

### CHK2 is involved in recombination-dependent arrest but does not regulate DSB levels

The CHK2 kinase is an effector of the ATM-signaling cascade in response to ionizing radiation [38]. In meiosis in several species, CHK2 function is required to efficiently activate the recombination-dependent checkpoint [9]. In mouse, CHK2 is required to arrest oocytes with unrepaired DSBs, although it has been suggested based on histological analysis that CHK2 does not play a similar role in spermatocytes [29]. To test for CHK2 involvement in recombination-dependent arrest in mouse spermatocytes, we analyzed *Trip13* ^*mod/mod*^ *Chk2* ^−/−^ mice.

**Fig 5:**
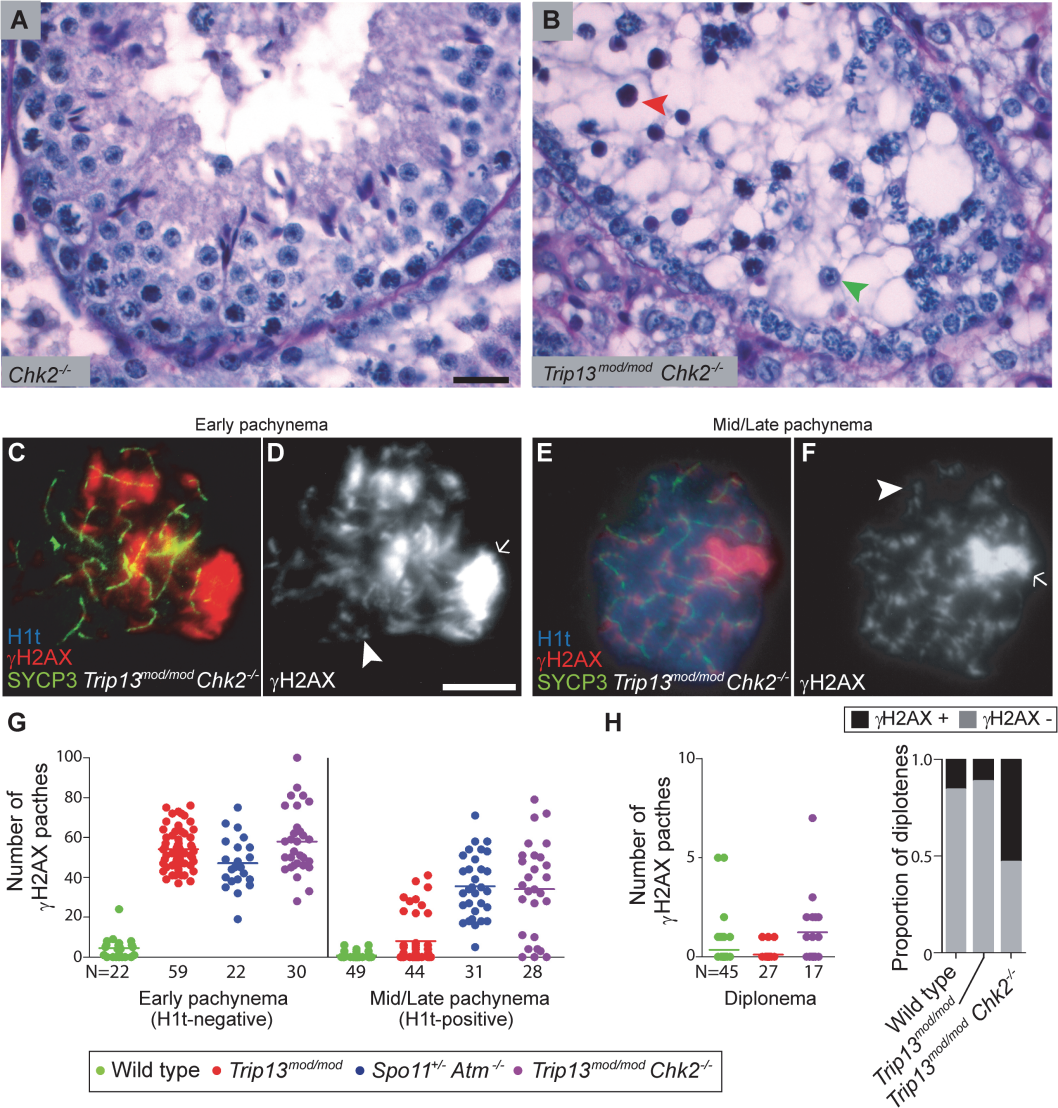
CHK2 is required to maintain early pachytene arrest caused by TRIP13 deficiency. (A-B) Cross-sections of *Chk2* ^−/−^ and *Trip13* ^*mod/mod*^ *Chk2* ^−/−^ testes stained with PAS-Haematoxylin. *Chk2* ^−/−^ testes contained all spermatogenic cell types, whereas *Trip13* ^*mod/mod*^ *Chk2* ^−/−^ spermatocytes arrested in tubules at epithelial stage IV (red arrowhead). Note the presence of spermatids (green arrowhead) indicating that some *Trip13* ^*mod/mod*^ *Chk2* ^−/−^ cells manage to complete meiosis. (C-F) Early and mid/late pachytene *Trip13* ^*mod/mod*^ *Chk2* ^−/−^ spermatocytes stained for H1t, γH2AX, and SYCP3. Both early and mid/late pachytene cells exhibit multiple γH2AX patches (arrowheads) corresponding to unrepaired DSBs, as well as accumulation of γH2AX on the chromatin of the sex chromosomes (arrows). (G) Quantification of the number of γH2AX patches in *Trip13* ^*mod/mod*^ *Chk2* ^−/−^ spermatocytes. Data for wild type, *Trip13* ^*mod/mod*^ and *Spo11* ^*+/−*^ *Atm* ^−/−^ reproduced from Fig. 1D for comparison, N shows the total number of cells counted per each stage and genotype. (H) γH2AX patches in diplotene cells. The plot on the left shows the number of γH2AX patches per cell. Note that overplotting of zero values obscures information about proportions of cells that lack a γH2AX patch, so the plot on the right displays proportions of cells with or without a γH2AX patch. Primary data for panels G and H are provided in S1 Dataset. N shows the total number of cells counted per each stage and genotype. Bar in (A) represents 20 μm and applies to panels (A-B). Bar in (D) represents 10 μm and applies to panels (C-F).

The testicular phenotype of *Trip13* ^*mod/mod*^ *Chk2* ^−/−^ mice was similar to the *Trip13* ^*mod/mod*^ single mutant for testis size and histological tubule classification (apoptosis of spermatocytes at epithelial stage IV, but with presence of some spermatids), but also, notably, for the percentage of SYCP3-expressing cells that had progressed beyond pachynema (1.9%, P = 0.2462, Fisher’s exact test; Table 1 and Fig. 5A-B). Thus, CHK2 deficiency does not cause a more penetrant spermatogenic failure in the context of the *Trip13* ^*mod/mod*^ mutation, unlike MRE11 complex hypomorphs or the absence of ATM itself. Moreover, CHK2-deficient testes displayed similar levels of SPO11-oligonucleotide complexes as wild-type littermates (Fig. 4D). Thus, CHK2 is not required for proper control of meiotic DSB formation, clearly separating CHK2 from both the MRE11 complex and ATM in the regulation of SPO11 activity. These results further support the conclusion above that the synaptic defects and more penetrant block to spermatogenesis observed in TSA triple mutants and in *Trip13* ^*mod/mod*^ *Mre11* ^*ATLD/ATLD*^ and *Trip13* ^*mod/mod*^ *Nbs1* ^*ΔB/ ΔB*^ double mutants are attributable to the increased DSB numbers.

Notably, however, *Trip13* ^*mod/mod*^ *Chk2* ^−/−^ spermatocytes showed evidence of a defect in recombination-dependent arrest, because two-fold more H1t-positive spermatocytes were observed than with the *Trip13* ^*mod/mod*^ single mutant (Table 1). Similarly to TSA triple mutants and the other double mutants described above, these H1t-positive spermatocytes presented more γH2AX patches (34.1 ± 23.3; P≤0.0001, t test; Fig. 5E-G). Also, whereas 77.3% of the H1t-positive *Trip13* ^*mod/mod*^ spermatocytes (i.e., those inferred to be relatively recombination-proficient escapers) had very few γH2AX patches (< 8 per cell), only 21.4% of cells from *Trip13* ^*mod/mod*^ *Chk2* ^−/−^ mice were in this category (P≤0.0001, Fisher’s exact test), with the majority of cells having many more γH2AX patches (Fig. 5G). By contrast, H1t-negative cells had similar numbers of γH2AX patches in both *Trip13* ^*mod/mod*^ single mutant and *Trip13* ^*mod/mod*^ *Chk2* ^−/−^ double mutant mice (P = 0.18, t test, Fig. 5G). These results suggest that the absence of CHK2 allows spermatocytes with numerous unrepaired DSBs to bypass the recombination-dependent arrest during pachynema, reminiscent of what occurs in oocytes [29]. If this is correct, we would also expect to find evidence of unrepaired DSBs in those spermatocytes that had escaped pachytene arrest entirely. Indeed, γH2AX patches were substantially more prevalent at diplonema in *Trip13* ^*mod/mod*^ *Chk2* ^−/−^ spermatocytes: only 11.1% of diplotene *Trip13* ^*mod/mod*^ cells had γH2AX patches, but 52.9% of the *Trip13* ^*mod/mod*^ *Chk2* ^−/−^ spermatocytes had one or more of these markers of unrepaired DSBs (P = 0.0045, Fisher’s exact test) (Fig. 5H). (Mean ± SD of 0.1 ± 0.3 for *Trip13* ^*mod/mod*^ (Fig. 1D) vs. 1.2 ± 1.8 (n = 17) for *Trip13* ^*mod/mod*^ *Chk2^–/–^*; P = 0.0005, negative binomial regression; S1 Dataset.) We conclude that CHK2, like the MRE11 complex and ATM, is required to activate the recombination-dependent arrest occurring in *Trip13* ^*mod/mod*^ spermatocytes.

### *Trip13* ^*mod/mod*^ *Chk2* ^*−/−*^ spermatocytes have sex body defects

Although CHK2 deficiency allowed more efficient progression of TRIP13-deficient cells to an H1t-positive stage, the overall spermatogenic failure was not alleviated, such that most *Trip13* ^*mod/mod*^ *Chk2* ^−/−^ spermatocytes still underwent apoptosis in tubules at epithelial stage IV (Fig. 5B). We therefore hypothesized that the sex bodies of *Trip13* ^*mod/mod*^ *Chk2* ^−/−^ spermatocytes may have subtle defects that impede their function. Indeed, most *Trip13* ^*mod/mod*^ *Chk2* ^*−/−*^ spermatocytes showed an abnormally elongated γsH2AX-positive sex body chromatin domain (57.8%, N = 45, Fig. 5E-F), unlike the condensed, rounded structure seen in most wild-type H1t-positive spermatocytes (Fig. 1F,H). In addition, the intensity of the sex-body γH2AX signal was reduced to about half the wild-type level on average in H1t-positive pachytene *Trip13* ^*mod/mod*^ *Chk2* ^−/−^ spermatocytes (S5 Fig.; P ≤0.0001, t test). Although most H1t-positive pachytene *Trip13* ^*mod/mod*^ cells had sex bodies with normal morphology, a significant minority also showed the abnormal elongated form (11.3%, n = 53, P≤0.0001, Fisher’s exact test) and sex body γH2AX intensity was reduced on average (P = 0.033; see S5 Fig.).

**Fig 6:**
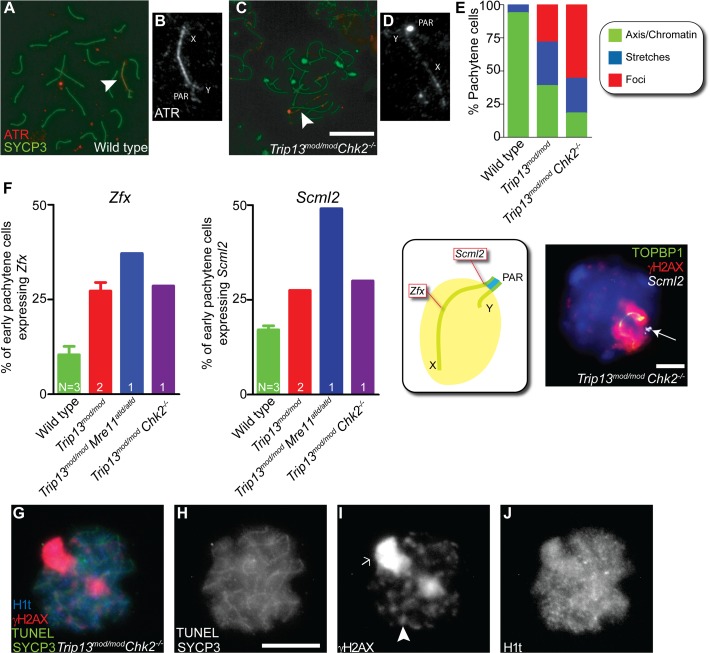
Sex body deficiency in *Trip13* mutants. (A-D) Wild-type and *Trip13* ^*mod/mod*^ *Chk2* ^−/−^ pachytene spermatocytes stained for ATR and SYCP3. Arrowheads indicate the sex chromosomes. Note the relatively continuous ATR staining on the X and Y axes in wild type compared with the focal ATR staining in the mutant. (E) Quantification of the different ATR staining patterns found in the genotypes presented. (F) Left panels, percent of early pachytene-stage spermatocytes expressing *Zfx* and *Scml2* in wild-type, *Trip13* ^*mod/mod*^, *Trip13* ^*mod/mod*^ *Mre11* ^*ATLD/ATLD*^ and *Trip13* ^*mod/mod*^ *Chk2* ^−/−^ mice. The N value in each bar represents the number of mice analyzed per each gene and genotype. Middle panel, cartoon of a sex body displaying the relative position of *Zfx* and *Scml2* within the X chromosome. Right image, representative *Trip13* ^*mod/mod*^ *Chk2* ^−/−^ spermatocyte displaying TOPBP1 (green) and γH2AX (red) immunostaining and *Scml2* RNA-FISH signal (white, arrow). Just 10.9% ± 3.8 (mean ± SD) of wild-type spermatocytes (N = 225 cells) express *Zfx* and 17.1% ± 1.9 express *Scml2* (N = 200). Mouse that fail to synapse the X and Y chromosomes, like *Trip13* ^*mod/mod*^ *Mre11* ^*ATLD/ATLD*^, present more spermatocytes expressing these genes than wild-type mice (for *Zfx*: 37.1%, N = 35 and for *Smcl2*: 49.1%, N = 53, P≤0.0001 and P = 0.0002 respectively, Fisher’s exact test). *Trip13* ^*mod/mod*^ mice present more cells expressing these genes than wild-type mice (for *Zfx*: 27.2% ± 3.2, N = 158; for *Smcl2*: 27.5% ± 0.0, N = 160, P<0.005, 1 way Anova). Similarly, *Trip13* ^*mod/mod*^ *Chk2* ^−/−^ spermatocytes are more likely to express these X-linked genes than wild-type cells (for *Zfx*: 28.6%, N = 70; for *Smcl2*: 30.0%, N = 80, P = 0.0217 and P = 0.0009 respectively, Fisher’s exact test). (G-J) Mid/late pachytene-stage, TUNEL-positive *Trip13* ^*mod/mod*^ *Chk2* ^−/−^ spermatocyte immunostained for SYCP3 (green, H), γH2AX (red, I) and H1t (blue, J). Note the presence of an elongated sex body (arrow), multiple γH2AX patches (arrowhead) and H1t in the chromatin of the apoptotic cell. Bars in (C) and (H) represent 10 μm and apply to panels (A,C) and (G-J), respectively. Bar in (F) represents 5 μm.

ATR is the kinase responsible for the bulk of H2AX phosphorylation in sex bodies [18,24,25]. In wild type, most pachytene spermatocytes showed ATR staining either as a continuous signal on the sex chromosome axes or spread to the XY chromatin (93.6%, n = 47; Fig. 6A-B), as previously reported [39]. The remainder of the cells displayed stretches of ATR partially covering the X and Y axes. By contrast, only 18.0% of *Trip13* ^*mod/mod*^ *Chk2* ^−/−^ pachytene cells had the axial or chromatin-associated ATR staining most commonly found in wild-type cells, 26.2% had short stretches of continuous ATR signal partly covering sex chromosome axes, and most cells had only focal ATR staining (55.7%, n = 61; Fig. 6C-E). *Trip13* ^*mod/mod*^ spermatocytes had an intermediate phenotype: 38.8% displayed ATR completely covering the XY axes or chromatin, 32.8% showed ATR stretches over the XY axes, and 28.4% had only discrete ATR foci along the X and Y (n = 67, Fig. 6E). These results suggest that CHK2 deficiency may exacerbate a defect in ATR loading on the sex chromosomes in TRIP13-deficient cells.

To further explore ATR-dependent processes, we examined SUMO-1, which is loaded onto sex chromosomes at pachynema in an ATR-dependent manner [18]. In all wild-type cells (n = 52) and 86.0% of *Trip13* ^*mod/mod*^ cells (n = 50), we detected SUMO-1 covering the chromatin of the sex body. In contrast, only 72.3% of *Trip13* ^*mod/mod*^ *Chk2* ^−/−^ pachytene spermatocytes contained SUMO-1 in their sex bodies, and of these, SUMO-1 signal had failed to spread over the entire XY chromatin in 60.6% of cells (n = 45, S6 Fig.).

Since the analyzed markers suggested that the sex bodies of *Trip13* ^*mod/mod*^ *Chk2* ^–/–^spermatocytes may be altered, we studied the functionality of these sex bodies by RNA FISH. As mentioned above, an important function of the sex body is to silence certain X- and Y-linked genes that are toxic for spermatocytes [17]. Thus, we analyzed the early-pachytene expression of two X-linked genes: *Zfx*, located in an interstitial region; and *Scml2*, located near the pseudoautosomal boundary at the centromere-distal end of the chromosome (Figs. 6F and S7). As expected, only a minority of wild-type cells expressed *Zfx* or *Smcl2* (10.9% ± 3.8 and 17.1% ± 1.9, mean ± SD, respectively, Fig. 6F), whereas a significantly larger fraction of cells expressed these genes in mutants that fail to form a sex body at pachynema, like *Trip13* ^*mod/mod*^ *Mre11* ^*ATLD/ATLD*^ spermatocytes (for *Zfx*: 37.1% and for *Smcl2*: 49.1%, P≤0.0001 and P = 0.0002 respectively, Fisher’s exact test). As predicted from the altered sex body morphology, we found that *Trip13* ^*mod/mod*^ and *Trip13* ^*mod/mod*^ *Chk2* ^−/−^ spermatocytes also showed increased expression of these X-linked genes at early pachynema (*Trip13* ^*mod/mod*^: *for Zfx*: 27.2% ± 3.2 and for *Smcl2*: 27.5% ± 0.0, P<0.005, 1 way Anova; *Trip13* ^*mod/mod*^ *Chk2* ^–/–^: *for Zfx*: 28.6% and for *Smcl2*: 30.0%, P = 0.0217 and P = 0.0009 respectively, Fisher’s exact test). We conclude that sex body function is altered in *Trip13* mutants.

The levels of sex chromosome gene misexpression are similar to those reported to be sufficient to arrest spermatocytes at pachynema in other mutants [21]. Indeed, when we assessed whether *Trip13* ^*mod/mod*^ *Chk2* ^−/−^ pachytene spermatocytes undergo apoptosis at an H1t-positive stage, as occurs in mutants that fail to form a sex body (S1 Fig.) [2], we found that 98.5% of TUNEL-positive *Trip13* ^*mod/mod*^ *Chk2* ^−/−^ spermatocytes analyzed were also H1t-positive, contrasting what occurs in *Trip13* ^*mod/mod*^ mutants (N = 67, 1 mouse, P ≤0.0001, Fisher’s exact test, Fig. 6G-J) but mimicking what is seen in *Spo11* ^−/−^ mutants (P = 1, Fisher’s exact test). In summary, the mild sex body defect in *Trip13* ^*mod/mod*^ testes was more pronounced in *Trip13* ^*mod/mod*^ *Chk2* ^−/−^ spermatocytes, where it resulted in the failure to silence the X and Y chromosomes at pachynema, suggesting that the stage IV apoptosis observed in these double mutant mice is triggered by the sex body-deficient arrest mechanism.

## Discussion

### Recombination-dependent arrest prior to H1t incorporation depends on the MRE11 complex, ATM and CHK2 in mouse

Two major mechanisms are postulated to mediate arrest of recombination-defective mouse spermatocytes during the pachytene stage [2]. One occurs when sex body formation is impeded by massive synaptic failure, irrespective of the presence of uncompleted recombination events, such as when no DSBs are generated at all [16]. Sex body defects allow expression of X and Y chromosome genes that are sufficient to induce apoptosis [17]. In this scenario, the arrest and later cell death might be caused by misregulation of gene expression, not activation of a checkpoint *per se*.

The other arrest mechanism is proposed to occur when recombination intermediates persist at pachynema [2,16], similar to what is seen in many other organisms [9]. In most species analyzed, ATR (Mec1 in budding yeast) activates this checkpoint, most likely due to the presence of single-stranded DNA [6]. However, recent observations in yeast suggest that Tel1 (ATM) might also be involved [40] and our findings clearly indicate that the ATM signaling cascade is crucial for recombination-dependent arrest in mammalian spermatocytes.

A recent study found that the previously described recombination-dependent arrest of *Atm* ^−/−^ oocytes [4] is mediated by CHK2 [29]. It was further suggested that a protein kinase other than ATM, possibly ATR, is involved in recombination-dependent arrest in oocytes [29]. While our results suggest that ATM is the principal kinase mediating this arrest in spermatocytes, we do not exclude the possibility that ATR also participates in this mechanism, either in wild type or specifically in *Atm^−/−^* cells, in the latter case by replacing some ATM functions (e.g., H2AX phosphorylation [24,25,41]).

In somatic cells, the MRE11 complex participates in ATM activation, and mutations that impair the MRE11 complex lead to an inefficient G2/M checkpoint [26]. NBS1 also specifically promotes ATM phosphorylation of some of its substrates [33]. Our findings show that recombination-dependent arrest at pachynema has similarities to the G2/M checkpoint in somatic cells, as previously speculated [42]. These similarities may be useful for identifying other key members of the meiotic pathway.

Involvement of the effector kinase CHK2 in mammalian recombination-dependent arrest, further supported by observations in oocytes [29], underlines differences between organisms [9]. In budding yeast, for instance, the checkpoint depends on the meiosis-specific CHK2 homolog, Mek1. In contrast, *D*. *melanogaster* uses the non-meiosis-specific CHK2 homolog, Mnk, and in *C*. *elegans* CHK1, but not CHK2, is required for arrest. This variety emphasizes the importance of studying mouse meiosis to characterize this pathway in mammals.

This and previous studies establish H1t expression as a molecular marker of a response to recombination defects in spermatocytes, and our current findings establish that this response occurs via an ATM signaling cascade. Although available data rule out substantially premature H1t expression, it is possible that H1t is expressed slightly earlier than normal in the absence of DSBs or in the absence of the ATM response; such an effect might contribute to the high observed fraction of spermatocytes that are H1t-positive in the relevant mutants. However, if so, we note that this would be consistent with our conclusion that regulation of H1t expression is a checkpoint response since there are many instances where cell cycle regulated events occur prematurely when either the monitored event or the monitoring mechanism are missing (e.g., yeast Ndt80 expression when DSBs or Mec1 signaling are absent [43] or anaphase onset when the spindle assembly checkpoint is deactivated [44]). It is formally possible that this response has little or no other consequence than the control of H1t expression, and that spermatocytes do not otherwise “care” that they have sensed the presence of unrepaired DSBs. We consider this hypothesis unlikely since it would mean that the male germline in mouse, unlike mouse oocytes and meiotic cells in all other species studied to date, ignores molecular signals that report directly on the progression of one of the most central aspects of meiotic chromosome dynamics (recombination), and ignores signals that have pronounced consequences in other mouse cell types. Instead, we favor the interpretation that the ATM-dependent response described here is an integral part of meiotic quality control surveillance during spermatogenesis.

### The recombination-dependent checkpoint is tolerant of a certain level of unrepaired DSBs

Our analysis exposed a population of *Trip13* ^*mod/mod*^ spermatocytes that were H1t-positive (i.e., had progressed to at least mid pachynema) even though they displayed as many as 41 γH2AX patches. These findings may suggest an overt defect in the recombination-dependent arrest mechanism or, alternatively, that this checkpoint tolerates a certain level of unrepaired DSBs. We favor the latter interpretation because the sex chromosomes in male meiocytes naturally accumulate DSBs during meiotic prophase, and recombination markers such as RAD51 can be observed on the X chromosome until late pachynema [45,46]. Since most DSBs formed on the X and Y chromosomes occur outside the pseudoautosomal region, they are presumably repaired by sister-chromatid recombination, which is thought to be permitted only later in pachynema [30]. Furthermore, in wild-type mice and in humans, many oocytes present multiple unrepaired DSBs at pachynema and even at diplonema [41,47]. Thus, we envision that recombination-surveillance mechanisms permit the progression of mammalian spermatocytes and oocytes that have sufficiently few DSBs to be compatible with eventual repair by the end of meiotic prophase and thus with long-term cell viability.

### TRIP13, MRE11 complex and ATM interplay to promote homologous chromosome synapsis

As previously stated, homologous chromosome synapsis depends on meiotic recombination [48]. Thus, failure to complete synapsis might be interpreted as a consequence of defective homologous recombination. Unlike the relevant single mutants, spermatocytes from TSA triple mutants and from *Trip13* ^*mod/mod*^ *Mre11* ^*ATLD/ATLD*^ and *Trip13* ^*mod/mod*^ *Nbs1* ^*ΔB/ΔB*^ double mutants fail to complete synapsis in all, or almost all, cells [22–25,36]. The MRE11 complex, ATM and TRIP13 have been reported to promote meiotic recombination [22–25,36]. Thus, the fact that double and triple mutants present problems with completing synapsis suggests that the MRE11 complex and ATM could synergize with TRIP13 to promote proper DSB repair during meiotic prophase. However, we provide evidence that proteins required to activate ATM (MRE11 and NBS1) are also involved in the regulation of DSB formation. Thus, since *Spo11* ^*+/−*^ *Atm* ^−/−^ spermatocytes as well as cells from *Mre11* ^*ATLD/ATLD*^ and *Nbs1* ^*ΔB/ΔB*^ single mutants incur more DSBs than wild-type cells and because TRIP13-deficient cells are unable to complete meiotic recombination [22,23], this opens the possibility that TRIP13-defective cells cannot deal with the additional DSBs formed in the absence, or reduction, of ATM activity. Alternatively or in addition, the synapsis phenotype observed in TSA triple mutants or *Trip13* ^*mod/mod*^ *Mre11* ^*ATLD/ATLD*^ and *Trip13* ^*mod/mod*^ *Nbs1* ^*ΔB/ΔB*^ double mutants might be a manifestation of a function of ATM and/or the MRE11 complex in promoting synapsis more directly and synergistically with TRIP13.

**Fig 7:**
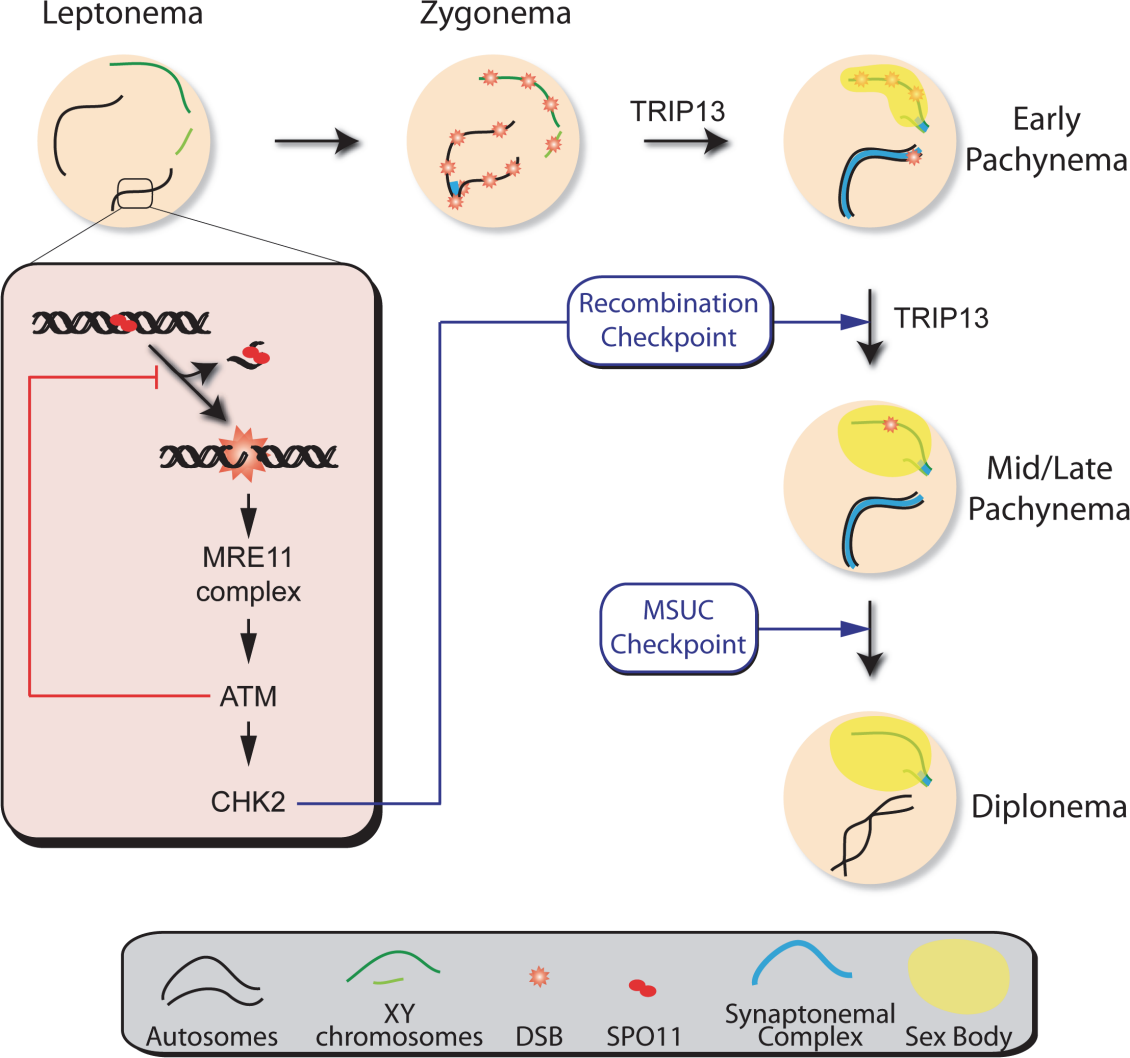
Functions of the ATM signaling pathway during mouse meiotic prophase. We propose that DSBs induced by SPO11 at the beginning of meiotic prophase are sensed by the MRE11 complex, which activates ATM. ATM inhibits further DSB formation via a feedback loop and promotes DSB repair. ATM activates CHK2, which controls cell cycle progression but is not involved in regulating DSB formation. Recombination progression leads to homologous chromosome synapsis and sex body formation. Besides participating in recombination, TRIP13 is also needed for effective sex body formation. Proper recombination and sex body formation are required to permit meiotic progression.

The array of phenotypes in the mutants analyzed here suggests that ATM participates in different aspects of meiotic prophase (Fig. 7). Our results further demonstrate that, while meiotic progression depends on the MRE11 complex, ATM and CHK2, the regulation of SPO11 activity requires the MRE11 complex to activate ATM but is independent of CHK2. This finding may indicate that direct ATM phosphorylation targets exert control of DSB formation, as has been argued in yeast [49]. Interestingly, yeast Mek1 promotes inter-homolog bias in the repair of DSBs [50,51]. The fact that homologous chromosome synapsis is not altered in the absence of mouse CHK2 suggests that this protein is not involved in recombination partner choice in mammals, although we cannot exclude that compensation by related kinases (e.g., CHK1) could be responsible for the absence of an obvious phenotype in *Chk2* ^−/−^ spermatocytes.

### TRIP13 participates in proper sex body formation

The apoptosis of spermatocytes in tubules at epithelial stage IV observed in *Trip13* ^*mod/mod*^ *Chk2* ^−/−^ testis can be explained if TRIP13 also contributes to the formation of a functional sex body (Fig. 7). In support of this hypothesis, we observed defective loading of ATR onto XY chromatin in pachytene spermatocytes from *Trip13* ^*mod/mod*^ *Chk2* ^−/−^ mice and in a subset of pachytene cells from *Trip13* ^*mod/mod*^ single mutants. This defect leads to inappropriate H2AX phosphorylation and SUMO-1 incorporation into the sex body, resulting in inefficient sex chromosome silencing and apoptosis at mid/late pachynema. TRIP13 is required for normal chromosomal association of HORMA (“Hop1, Rev7 and Mad2”)-domain proteins HORMAD1 and HORMAD2 during meiotic prophase [52]. HORMAD1 and HORMAD2 localize to the chromosome axes at leptonema and disappear from synapsed regions during zygonema as synapsis progresses. At pachynema, HORMAD1 and HORMAD2 accumulate at the unsynapsed regions of the X and Y chromosomes where they attract the machinery required to silence these chromosomes [21,53]. In *Trip13* ^*mod/mod*^ spermatocytes, HORMAD1 and HORMAD2 are retained in the synapsed regions of chromosomes and are present on all bivalent axes at pachynema [52]. We speculate that this aberrant localization of HORMA-domain proteins may contribute to inefficient ATR loading on sex chromosomes of *Trip13* ^*mod/mod*^ *Chk2* ^−/−^ pachytene spermatocytes. It is worth noting that *Trip13* ^*mod/mod*^ *Chk2* ^−/−^ cells do assemble ATR foci, presumably at resected DSB sites containing single-stranded DNA. This focal ATR localization has been reported previously to be HORMAD2-independent [21]. However, loading of ATR along the entire length of the unsynapsed chromosome axis is HORMAD2-dependent [21,53]. Furthermore, *Zfx* and *Scml2* expression levels in early pachytene-stage *Trip13* mutant spermatocytes are similar to those reported in *Hormad2* ^−/−^ cells [21]. These findings lead us to propose that proper HORMAD1 and HORMAD2 localization may underlie the function of TRIP13 in sex body formation.

The unexpected failure to properly form a sex body found in *Trip13* mutants opens the possibility that, although this and other studies have clearly shown that there are at least two distinct arrest mechanisms to respond to recombination or sex body defects [2,16], the apoptosis occurring after the activation of these two arrest mechanisms may be a response only to the common MSCI failure. Nonetheless, we favor the hypothesis that recombination-dependent arrest is sufficient to trigger apoptosis because unrepaired DSBs provoke programmed cell death in oocytes (where MSCI defects are not an issue) via the conventional DNA damage response effector molecules, p53 and p63 [4,22,29]; and because MRE11- and ATM-dependent signaling can directly promote apoptosis in somatic cells [26]. Addressing this issue will require novel meiotic mutants that display recombination defects in the context of an intact ATM signaling cascade and without MSCI defects.

## Materials and Methods

### Mutant mice

*Trip13*, *Spo11*, *Atm*, *Dmc1*, *Mre11*, *Nbs1* and *Chk2* mutations were generated previously [10,14,23,28,34,35,54]. All lines were maintained on a C57BL6–129Sv mixed background. Experiments were performed using at least two animals in comparison with littermate controls (either homozygous or heterozygous for the wild-type alleles) or, when appropriate littermates were unavailable, control animals obtained from litters of the same matings. Genotyping was performed by PCR analysis of tail-tip DNA as previously described [23].

### Cytology and histology

Testes were harvested from two- to five-month-old animals and processed for histology or cytology, as previously described [23,55]. Immunofluorescence was performed using standard methods [47] and antibodies [23]. Additional primary antibodies used were: guinea pig anti-H1t (kindly donated by M.A. Handel, Jackson Lab) at 1:500 dilution [12] and a mouse anti-SUMO-1 (clone 21C7, Invitrogen) at 1:100 dilution. TUNEL-staining on IF-stained slides was performed using an *in situ* cell death detection kit (Roche Diagnostics) according to the manufacturer’s instructions, as reported previously [56].

### RNA FISH and immunofluorescence

RNA FISH was carried out with digoxigenin-labeled probes as previously described [57]. BAC DNA probes used were: *Zfx*, bMQ-372M23 (from Mouse bMQ BAC library) and *Scml2*, RP24-204O18 (from CHORI BACPAC library). Briefly, BAC-containing bacteria were grown in an overnight LB-Chloramphenicol culture at 37°C and BAC DNA was isolated using a standard miniprep method. Approximately 2 μg of BAC DNA was labelled using DIG-Nick Translation Mix (Roche) and precipitated with Cot-1 DNA (Invitrogen) and salmon sperm DNA (Stratagene). Mouse testes were minced and cells were permeabilized with CSK buffer (100 mM NaCl, 300 mM sucrose, 3 mM MgCl_2_, 10 mM PIPES, 0.5% Triton X-100, 2 mM vanadyl ribonucleoside (New England Biolabs)), fixed with 4% paraformaldehyde and dehydrated through an ice-cold ethanol series. DNA-BAC probes were denatured for 10 min at 80°C, pre-hybridized for 30 min at 37°C, and added to the slides for an overnight incubation at 37°C. Stringency washes were performed and digoxigenin was detected using anti-digoxigenin-FITC (1:10, Millipore). RNA FISH was then followed by TOPBP1 (1:50, Abcam) and γH2AX (1:100, Millipore) immunostaining. Cells were examined on an Olympus IX70 inverted microscope. Images were captured using a computer-assisted (DeltaVision) CCD camera (Photometrics), and processed for publication using ImageJ and Photoshop. Early pachytene cells were defined based on a continuous TOPBP1 staining along the X and Y chromosome axes.

### SPO11-oligonucleotide complex detection and Western blotting

Testis extract preparation, immunoprecipitation and Western blot analysis were performed essentially as described [27]. SPO11-oligonucleotide complexes and free SPO11 were isolated from testis lysates by two rounds of immunoprecipitation with a SPO11 monoclonal antibody (Spo11–180) on Protein A-agarose beads (Roche). SPO11-oligonucleotide complexes were labeled with [α-^32^P] dCTP using terminal deoxynucleotidyl transferase (Fermentas), released from the beads by boiling in Laemmli buffer, and fractionated by SDS-PAGE. The electrophoresed products were transferred onto polyvinylidene fluoride (PVDF) membrane. Radiolabeled species were detected using Fuji phosphor screens and quantified with ImageGauge software. The same PVDF membrane was then subjected to Western analysis using the SPO11 monoclonal antibody.

### Statistical analysis

One-way ANOVA, Student's t tests and Fisher's exact tests were performed using GraphPad Prism software and/or GraphPad QuickCalcs online resource (http://www.graphpad.com/quickcalcs/). For comparing counts of γH2AX foci, we used t tests for simplicity to compare between genotypes at pachynema because the count data were not highly skewed (i.e., were reasonably approximated by a normal distribution). However, for diplotene cells we instead used negative binomial regression because the count distributions at this stage were highly skewed for the *Trip13* ^*mod/mod*^ *Chk2* ^*−/−*^ sample and contained many zero values for all samples. Regression was carried out using the glm.nb function from the MASS package (version 7.3–33) in R (version 3.1.1).

### Ethics statement

All experiments performed in this study comply with US and EU regulations and were approved by the MSKCC Institutional Animal Care and Use Committee and the UAB Ethics Committee and the Catalan Government.

## Supporting Information

S1 Fig *Dmc1* ^−/−^ spermatocytes arrest at early pachynema and *Spo11* ^−/−^ spermatocytes arrest at mid/late pachynema. *Spo11* ^–/–^(A,C,E,G) and *Dmc1* ^–/–^(B,D,F,H) spermatocyte spreads were immunostained for H1t, γH2AX and SYCP3, and subjected to TUNEL staining as well. (H) The cell on the right side of the panel presents pan-nuclear staining characteristic of the TUNEL reaction (also seen in G). One mouse analyzed per genotype. Bar in (G) represents 10 μm and applies to all panels.
(TIF)

S2 Fig Triple mutant spermatocytes accumulate H1t. Representative wild-type (A-D), *Trip13* ^*mod/mod*^ (E-H) and *Trip13* ^*mod/mod*^ *Spo11* ^*+/−*^ *Atm* ^–/–^ (I-L) testis sections immunostained for H1t (green) and γH2AX (red). Note the significant increase in the number of spermatocytes positive for H1t in the triple mutant section compared to the *Trip13* ^*mod/mod*^ sample. Bar in (A) represents 10 μm and applies to panels A, E and I.
(TIF)

S3 Fig Timing of H1t incorporation into the chromatin of TSA triple mutant spermatocytes is not detectably altered compared to wild type. Individual panels in (A) and (C) show representative seminiferous tubule sections of the indicated epithelial stages from wild-type and *Trip13* ^*mod/mod*^ *Spo11* ^*+/−*^ *Atm* ^−/−^ testis sections immunostained for H1t (green) and γH2AX (red) and stained with DAPI to detect DNA. Insets on the right of each panel show higher magnification images of individual fluorescence channels for the cells boxed in white. Panels are ordered to provide a developmental timeline through prophase I. Examples of specific cell types are indicated: Sg, spermatogonia; Sp, spermatocyte (further subdivided into leptotene (L-Sp), zygotene (Z-Sp), early pachytene (EP-Sp), mid pachytene (MP-Sp), late pachytene (LP-Sp), and diplotene (D-Sp)); Sd, spermatid; Se, Sertoli cell. (A, B) H1t in wild type. Seminiferous tubule staging is based on the array of different cell types contained in a tubule section, with morphological differences regarding particular organelles used as markers to more precisely distinguish between cellular subtypes [19]. Tubule staging is more challenging using only the information obtained by immunofluorescence analysis of chromatin proteins, as compared to the histological staining methods traditionally used. Nonetheless, unambiguous staging can be performed in wild type testis, which contains all spermatogenic cell types. Leptotene spermatocytes make up the outermost layer of cells in stage IX and X tubules, with a layer of late pachytene cells and a layer of spermatids beginning to elongate their nuclei located more centrally toward the tubule lumen. Zygotene cells are in the outer layer of stage XI-XII tubules, which are further characterized for the presence of spermatids with a clearly elongated head. The outer layer of stage I tubules contains early pachytene cells that show the first manifestation of a stretched sex body; these tubules also contain two kinds of spermatids, round and elongated. From stage II-III onwards, spermatogonia for the next wave of spermatogenesis have proliferated to sufficient numbers to constitute a distinct basal layer of cells, so spermatocytes are now located in the second layer of cells. In stage II-III tubules, the outer layer of spermatogonia is followed by a layer of H1t-negative pachytene spermatocytes with a more condensed sex body, then a layer of H1t-positive round spermatids, and finally elongated spermatids. Stage IV tubules are similar, with the important exception that the pachytene spermatocytes are now clearly H1t positive. Stage IV can be clearly distinguished from other stages by examining the round spermatids: this stage is characterized by the presence of the acrosomal granules that begin to form the acrosomal vesicle on top of the nuclear envelope of round spermatids. This creates a small, rounded depression (angle < 40°) in the nuclei of these cells that clearly marks this stage (see (B) for a magnified image of the spermatids boxed in yellow in the stage IV tubule section). These results demonstrate that H1t protein expression first becomes detectable in mid-pachytene spermatocytes in stage IV tubules, consistent with prior studies [13]. (C) H1t expression timing is not grossly altered in the absence of ATM. Accurate classification of individual tubule stages is more difficult in mutants (including the TSA triple mutant) that experience spermatogenic arrest, because these mutants lack the post-meiotic cell types that facilitate a clear differentiation between stages [19]. Nonetheless, we can conclude that H1t expression does not begin until after the earliest part of pachynema in the TSA triple mutant, because spermatocytes were H1t-negative in all analyzed sections for which spermatocytes made up the outermost layer (i.e., corresponding to leptotene, zygotene or early pachytene spermatocytes from tubule stages IX-XII or stage I). In tubules in which spermatocytes made up the second layer of cells (i.e., stages II-IV), we observed that these pachytene spermatocytes were H1t-negative in some tubules and H1t-positive in others. In tubules with H1t-positive pachytene cells, we also frequently observed apoptotic figures (arrowheads in bottom-most panel), so we infer these are stage IV tubules. We cannot distinguish with absolute certainty between these tubule stages, so we cannot exclude the possibility that H1t expression begins slightly earlier than in wild type (e.g., in stage III, or earlier in stage IV than would normally occur), but the data are fully consistent with the hypothesis that H1t expression occurs with similar timing in the TSA triple mutant as in wild type.
(TIF)

S4 Fig *Trip13* ^*mod/mod*^ *Mre11* ^*ATLD/ATLD*^ spermatocytes arrest in mid/late pachynema with unrepaired DSBs at epithelial stage IV. (A-B) Cross-sections of *Mre11* ^*ATLD/ATLD*^ and *Trip13* ^*mod/mod*^ *Mre11* ^*ATLD/ATLD*^ stage IV tubules stained with PAS-Haematoxylin. (C-D) Early (C) and mid/late (D) pachytene *Trip13* ^*mod/mod*^ *Mre11* ^*ATLD/ATLD*^ spermatocytes stained for H1t, γH2AX, and SYCP3. Both cells present multiple unrepaired DSBs and incomplete synapsis. Bar in (A) represents 20 μm and applies to panels (A-B). Bar in (D) represents 10 μm and applies to panels (C-D).
(TIF)

S5 Fig TRIP13 is needed to properly phosphorylate H2AX on the chromatin of the sex chromosomes. Anti-γH2AX immunofluorescence intensity (arbitrary units) was measured on the sex bodies of mid/late pachytene spermatocytes of the indicated genotypes. Black horizontal bars represent the means.
(TIF)

S6 Fig TRIP13 is required to load SUMO-1 onto sex-chromosome chromatin at pachynema. Wild-type (A-D), *Trip13* ^*mod/mod*^ (E-H), and *Trip13* ^*mod/mod*^ *Chk2* ^–/–^(I-L) pachytene spermatocytes were immunostained for SUMO-1 and SYCP3 and counterstained with DAPI. For each cell, enlarged images of the sex chromosomes are provided. SUMO-1 signal covers the entire chromatin of the sex chromosomes in wild type and *Trip13* ^*mod/mod*^, but only occupies a portion of the sex-chromosome chromatin in *Trip13* ^*mod/mod*^ *Chk2* ^–/–^. Bar in (I) represents 10 μm and applies to panels (A,E and I).
(TIF)

S7 Fig *Scml2* is expressed in Trip13 mutant early pachytene spermatocytes. (A-L) Early pachytene spermatocytes from wild type (A-D), *Trip13* ^*mod/mod*^ (E-H), and *Trip13* ^*mod/mod*^ *Chk2* ^–/–^(I-L) immunostained against γH2AX and TOPBP1 and hybridized with an anti-*Scml2* probe. Positive RNA-FISH signals are pointed by an arrow (E and I).
(TIF)

S1 Table Summary of the most relevant aspects of the phenotypes of the different single, double and triple mutants used in this study available at the outset of this research and complemented with the results obtained in this study. Results obtained in this study are presented in italics. n.d., not determined.
(DOCX)

S1 Dataset Primary data (counts of γH2AX patches per cell) for the plots in Figs. 1D, 2J, 5G, 5H. (XLSX)
