# Supplementary figures and images for: The ATM Signaling Cascade Promotes Recombination-Dependent Pachytene Arrest in Mouse Spermatocytes
Source: PLoS Genet. 2015 Mar 13;11(3):e1005017. doi: 10.1371/journal.pgen.1005017 (PMC4358828; doi:10.1371/journal.pgen.1005017)

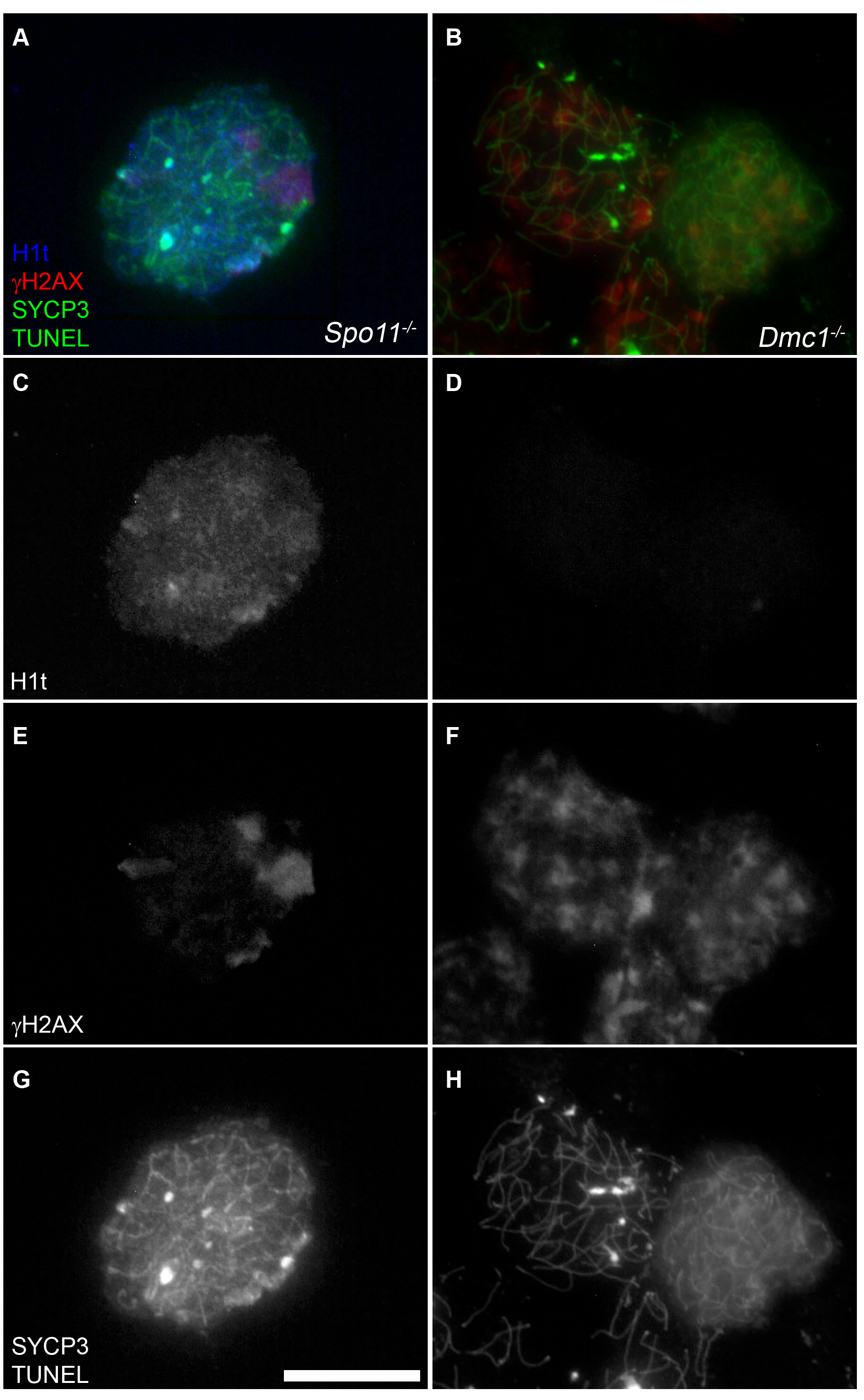

Supplement: S1 Fig — Spo11 –/–(A,C,E,G) and Dmc1 –/–(B,D,F,H) spermatocyte spreads were immunostained for H1t, γH2AX and SYCP3, and subjected to TUNEL staining as well. (H) The cell on the right side of the panel presents pan-nuclear staining characteristic of the TUNEL reaction (also seen in G). One mouse analyzed per genotype. Bar in (G) represents 10 μm and applies to all panels. (TIF) [file pgen.1005017.s001.tif]

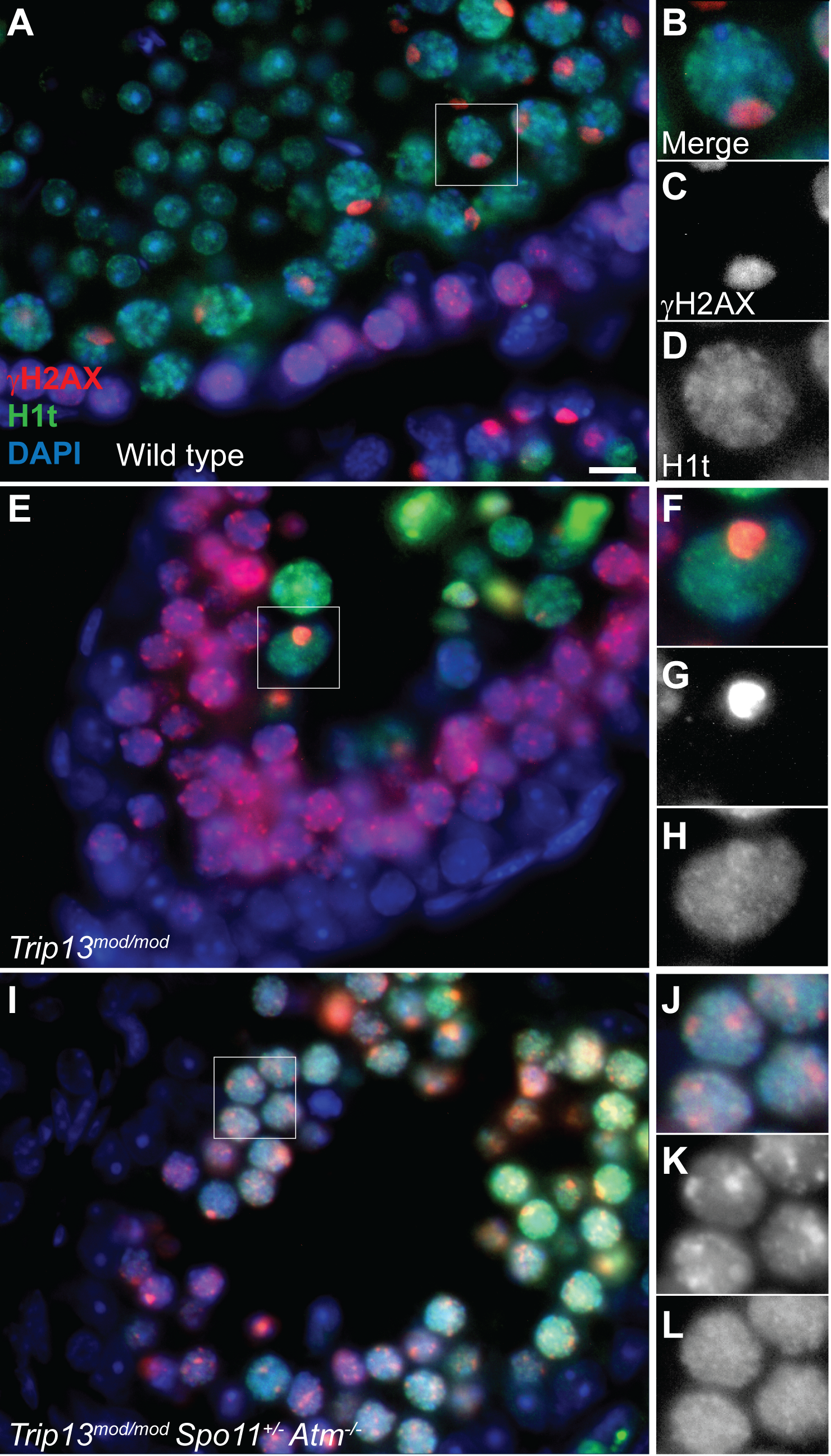

Supplement: S2 Fig — Representative wild-type (A-D), Trip13 mod/mod (E-H) and Trip13 mod/mod Spo11 +/− Atm –/– (I-L) testis sections immunostained for H1t (green) and γH2AX (red). Note the significant increase in the number of spermatocytes positive for H1t in the triple mutant section compared to the Trip13 mod/mod sample. Bar in (A) represents 10 μm and applies to panels A, E and I. (TIF) [file pgen.1005017.s002.tif]

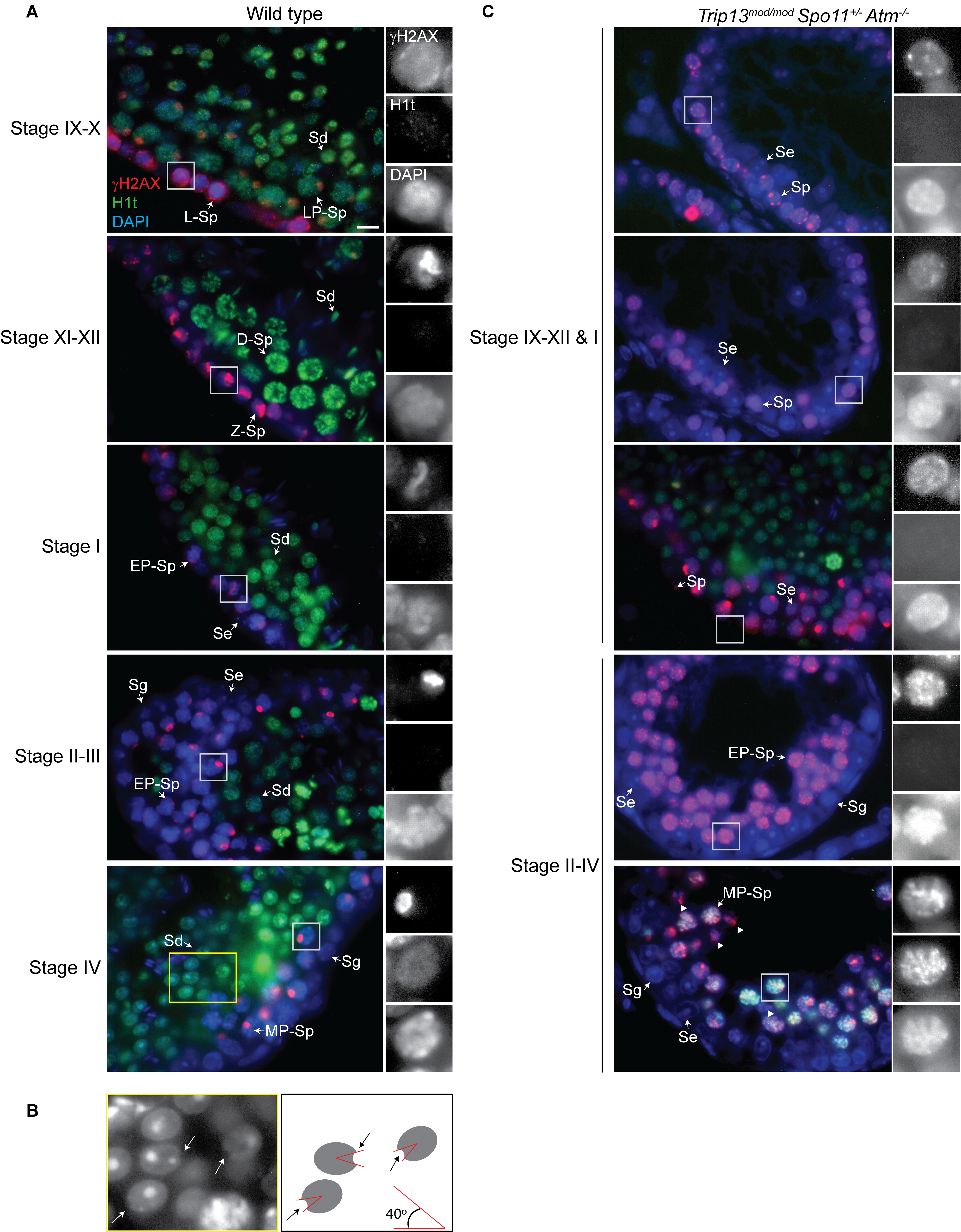

Supplement: S3 Fig — Individual panels in (A) and (C) show representative seminiferous tubule sections of the indicated epithelial stages from wild-type and Trip13 mod/mod Spo11 +/− Atm −/− testis sections immunostained for H1t (green) and γH2AX (red) and stained with DAPI to detect DNA. Insets on the right of each panel show higher magnification images of individual fluorescence channels for the cells boxed in white. Panels are ordered to provide a developmental timeline through prophase I. Examples of specific cell types are indicated: Sg, spermatogonia; Sp, spermatocyte (further subdivided into leptotene (L-Sp), zygotene (Z-Sp), early pachytene (EP-Sp), mid pachytene (MP-Sp), late pachytene (LP-Sp), and diplotene (D-Sp)); Sd, spermatid; Se, Sertoli cell. (A, B) H1t in wild type. Seminiferous tubule staging is based on the array of different cell types contained in a tubule section, with morphological differences regarding particular organelles used as markers to more precisely distinguish between cellular subtypes [19]. Tubule staging is more challenging using only the information obtained by immunofluorescence analysis of chromatin proteins, as compared to the histological staining methods traditionally used. Nonetheless, unambiguous staging can be performed in wild type testis, which contains all spermatogenic cell types. Leptotene spermatocytes make up the outermost layer of cells in stage IX and X tubules, with a layer of late pachytene cells and a layer of spermatids beginning to elongate their nuclei located more centrally toward the tubule lumen. Zygotene cells are in the outer layer of stage XI-XII tubules, which are further characterized for the presence of spermatids with a clearly elongated head. The outer layer of stage I tubules contains early pachytene cells that show the first manifestation of a stretched sex body; these tubules also contain two kinds of spermatids, round and elongated. From stage II-III onwards, spermatogonia for the next wave of spermatogenesis have [file pgen.1005017.s003.tif]

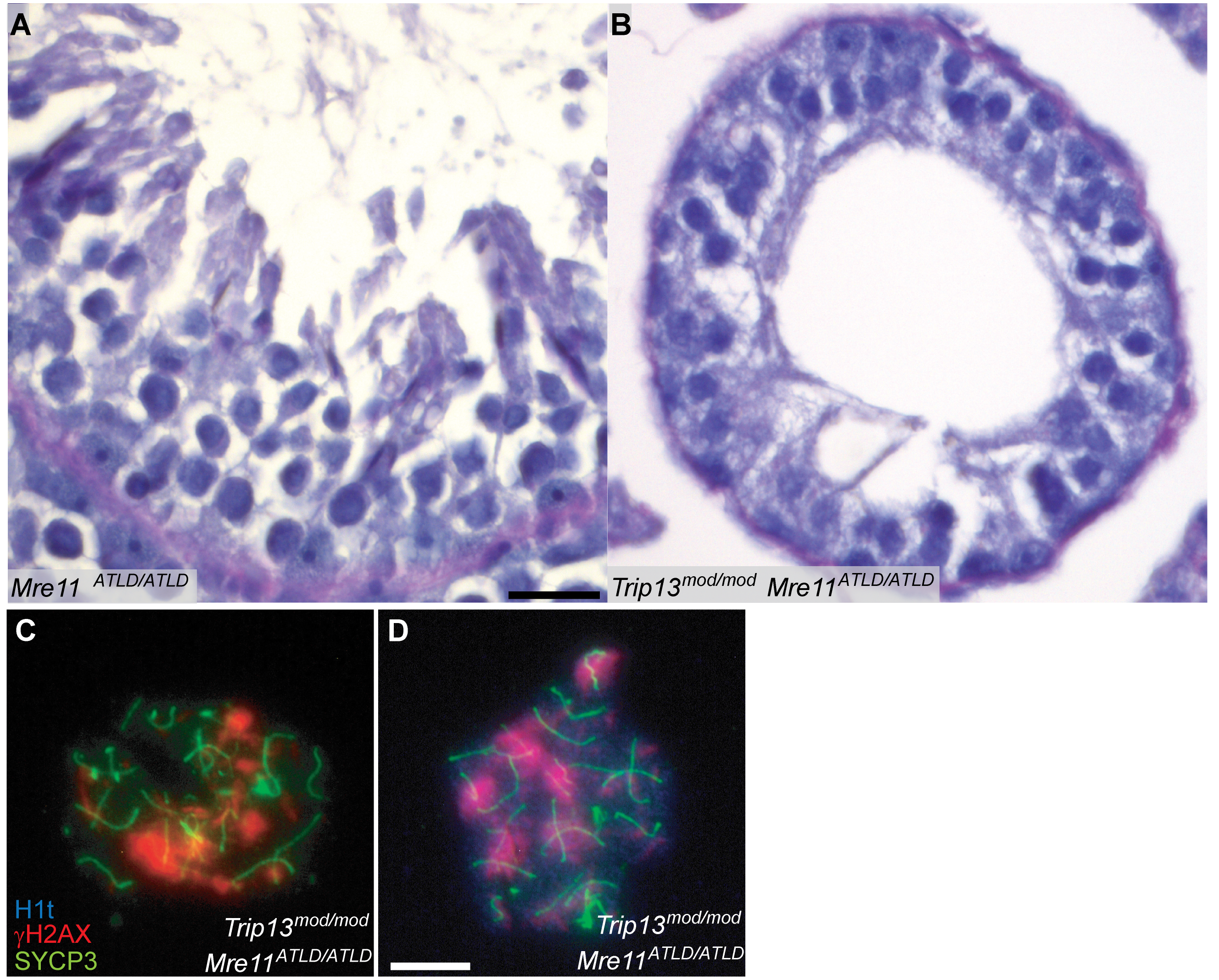

Supplement: S4 Fig — (A-B) Cross-sections of Mre11 ATLD/ATLD and Trip13 mod/mod Mre11 ATLD/ATLD stage IV tubules stained with PAS-Haematoxylin. (C-D) Early (C) and mid/late (D) pachytene Trip13 mod/mod Mre11 ATLD/ATLD spermatocytes stained for H1t, γH2AX, and SYCP3. Both cells present multiple unrepaired DSBs and incomplete synapsis. Bar in (A) represents 20 μm and applies to panels (A-B). Bar in (D) represents 10 μm and applies to panels (C-D). (TIF) [file pgen.1005017.s004.tif]

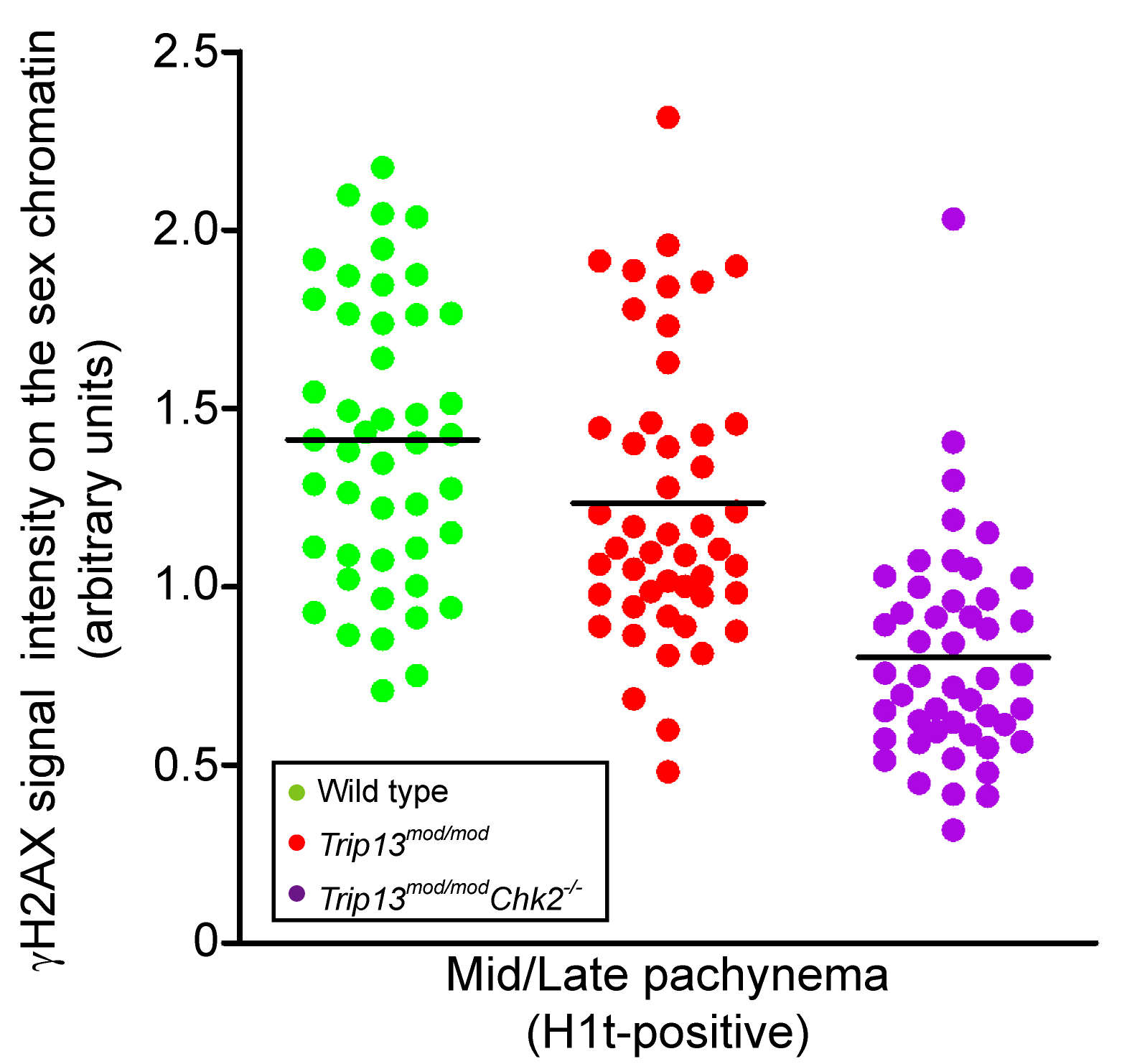

Supplement: S5 Fig — Anti-γH2AX immunofluorescence intensity (arbitrary units) was measured on the sex bodies of mid/late pachytene spermatocytes of the indicated genotypes. Black horizontal bars represent the means. (TIF) [file pgen.1005017.s005.tif]

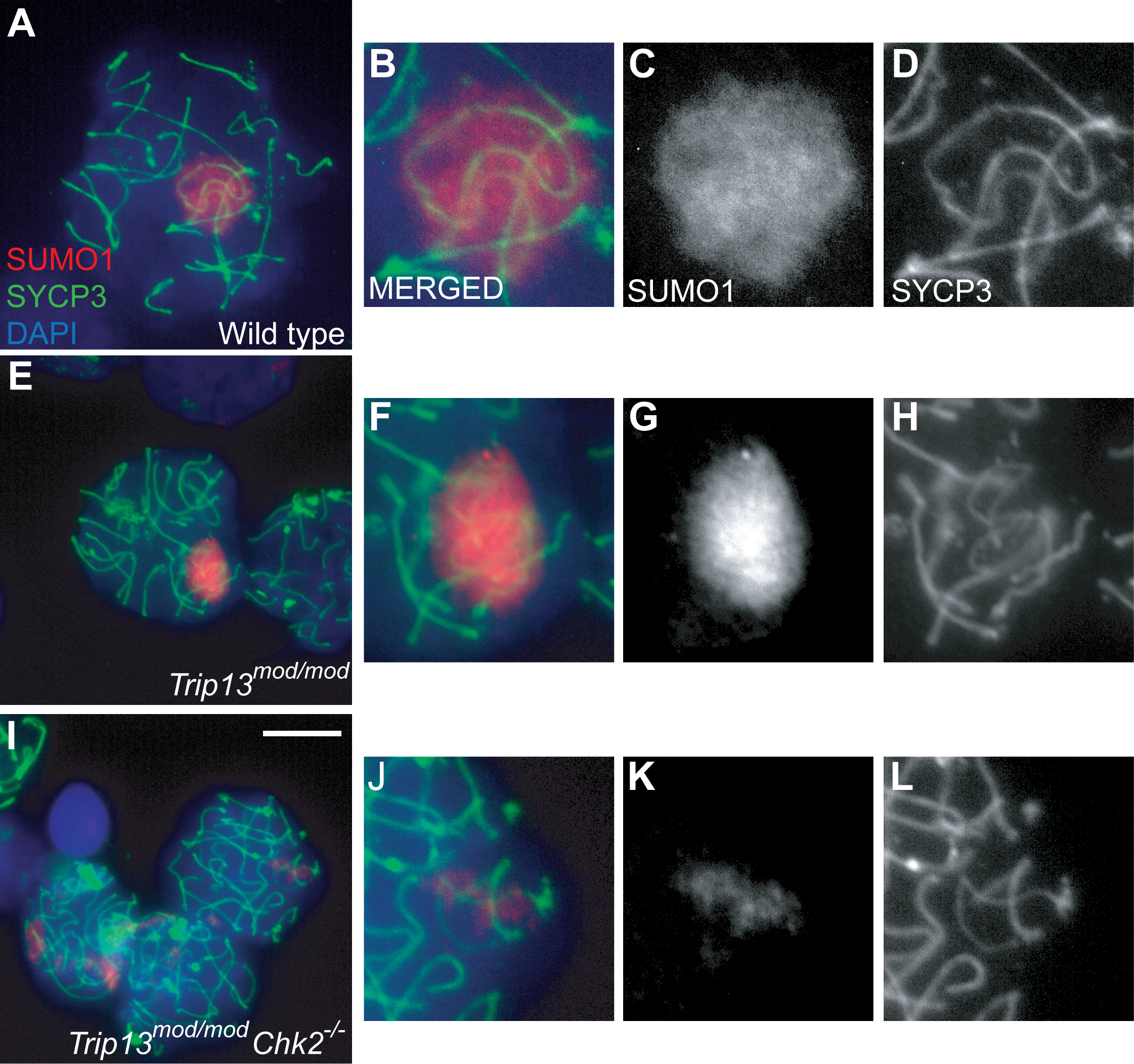

Supplement: S6 Fig — Wild-type (A-D), Trip13 mod/mod (E-H), and Trip13 mod/mod Chk2 –/–(I-L) pachytene spermatocytes were immunostained for SUMO-1 and SYCP3 and counterstained with DAPI. For each cell, enlarged images of the sex chromosomes are provided. SUMO-1 signal covers the entire chromatin of the sex chromosomes in wild type and Trip13 mod/mod, but only occupies a portion of the sex-chromosome chromatin in Trip13 mod/mod Chk2 –/–. Bar in (I) represents 10 μm and applies to panels (A,E and I). (TIF) [file pgen.1005017.s006.tif]

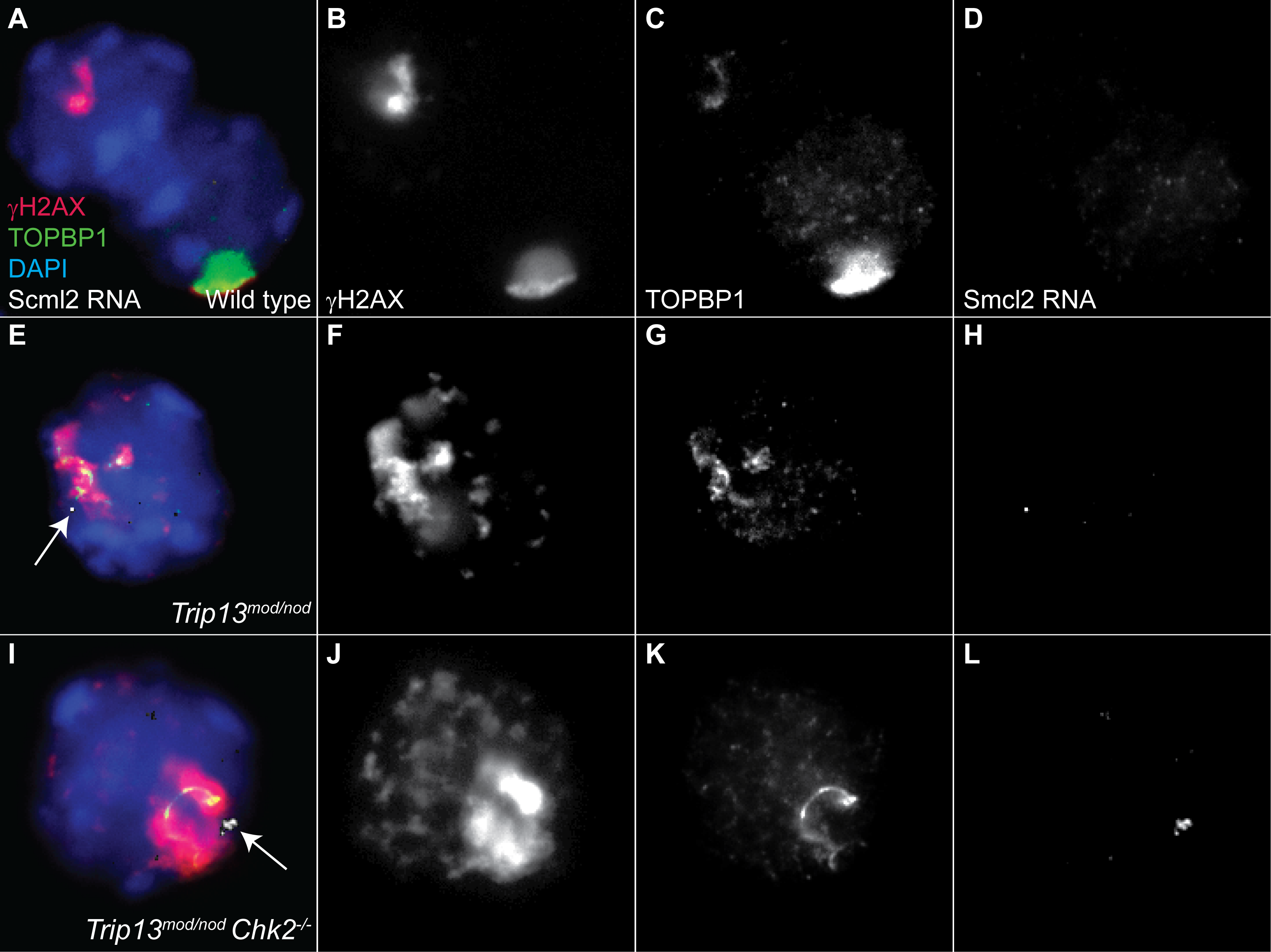

Supplement: S7 Fig — (A-L) Early pachytene spermatocytes from wild type (A-D), Trip13 mod/mod (E-H), and Trip13 mod/mod Chk2 –/–(I-L) immunostained against γH2AX and TOPBP1 and hybridized with an anti-Scml2 probe. Positive RNA-FISH signals are pointed by an arrow (E and I). (TIF) [file pgen.1005017.s007.tif]
