# Supplementary material for: The ATM Signaling Cascade Promotes Recombination-Dependent Pachytene Arrest in Mouse Spermatocytes
Source: PLoS Genet. 2015 Mar 13;11(3):e1005017. doi: 10.1371/journal.pgen.1005017 (PMC4358828; doi:10.1371/journal.pgen.1005017)
Supplement: S1 Table — Results obtained in this study are presented in italics. n.d., not determined. (DOCX) [file pgen.1005017.s008.docx]

| **Genotype** | **Fertility** | **Epithelial Arrest** | **H1t incorporation** | **Sex body formation** | **SPO11 function** | **Meiotic Recombination** | **Synapsis** | **References** |
| --- | --- | --- | --- | --- | --- | --- | --- | --- |
| *Atm*^–/–^ | Sterile | IV | Deficient | Deficient | Augmented (12x) | Deficient | Deficient | [2,24,53] |
| *Spo11*^+/–^ | Fertile | No arrest | n.d. | Proficient | Decreased | Proficient | Proficient | [21,54] |
| *Spo11*^+/–^ *Atm*^–/–^ | Sterile | XII (escapers) | *Proficient* | Proficient | Augmented (6x) | Deficient | Proficient | [21,24] and this study |
| *Spo11*^–/–^ | Sterile | IV | Proficient | Deficient | Nonexistent | Deficient | Deficient | [2,14,15] |
| *Dmc1*^–/–^ | Sterile | IV | Deficient | Deficient | As wild type | Deficient | Deficient | [2,13] |
| *Trip13^mod/mod^* | Sterile | IV (escapers) | *Deficient* | *Deficient* | *As wild type* | Deficient | Proficient | [19,20] and this study |
| *Mre11^ATLD/ATLD^* | Subfertile | No arrest | *Proficient* | Proficient | *Augmented (2x)* | Mildly deficient | Mildly deficient | [33] and this study |
| *Nbs1^ΔB/ΔB^* | Subfertile | No arrest | *Proficient* | Proficient | *Augmented (2x)* | Mildly deficient | Mildly deficient | [33] and this study |
| *Chk2*^–/–^ | Fertile | No arrest | *Proficient* | *Proficient* | *As wild type* | *Proficient* | *Proficient* | [49] and this study |
| *Trip13^mod/mod^ Spo11*^+/–^ *Atm*^–/–^ | *Sterile* | *IV* | *Proficient* | *Deficient* | *n.d.* | *Deficient* | *Deficient* | This study |
| *Trip13^mod/mod^ Mre11^ATLD/ATLD^* | *Sterile* | *IV* | *Proficient* | *Deficient* | *n.d.* | *Deficient* | *Deficient* | This study |
| *Trip13^mod/mod^ Nbs1^ΔB/ΔB^* | *Sterile* | *IV* | *Proficient* | *Deficient* | *n.d.* | *Deficient* | *Deficient* | This study |
| *Trip13^mod/mod^ Chk2*^–/–^ | *Sterile* | *IV (escapers)* | *Proficient* | *Deficient* | *n.d.* | *Deficient* | *Proficient* | This study |
